# Supplementary material for: Mutations in REEP6 Cause Autosomal-Recessive Retinitis Pigmentosa
Source: Am J Hum Genet. 2016 Nov 23;99(6):1305–15. doi: 10.1016/j.ajhg.2016.10.008 (PMC5142109; doi:10.1016/j.ajhg.2016.10.008)
Supplement: Document S1. Figures S1–S3 and Tables S1–S3 [file mmc1.pdf]

The American Journal of Human Genetics, Volume *99*

Supplemental Data

Mutations in *REEP6* Cause Autosomal-Recessive Retinitis Pigmentosa

Gavin Arno, Smriti A. Agrawal, Aiden Eblimit, James Bellingham, Mingchu Xu, Feng Wang, Christina Chakarova, David A. Parfitt, Amelia Lane, Thomas Burgoyne, Sarah Hull, Keren J. Carss, Alessia Fiorentino, Matthew J. Hayes, Peter M. Munro, Ralph Nicols, Nikolas Pontikos, Graham E. Holder, UKIRDC, Chinwe Asomugha, F. Lucy Raymond, Anthony T. Moore, Vincent Plagnol, Michel Michaelides, Alison J. Hardcastle, Yumei Li, Catherine Cukras, Andrew R. Webster, Michael E. Cheetham, and Rui Chen

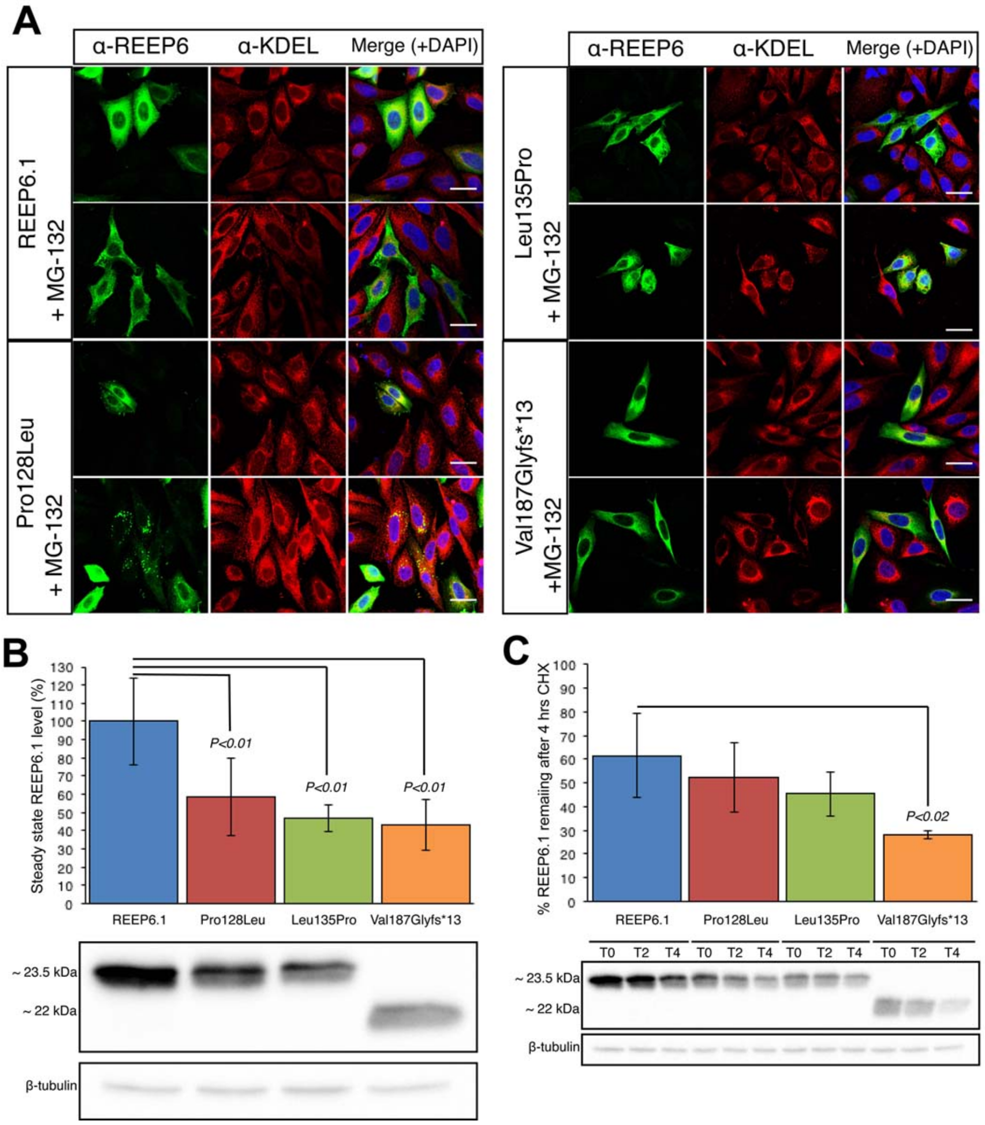

Figure S1. Variants in REEP6.1 affect protein stability. (A) The expression of REEP6.1 WT and the Pro128Leu, Leu135Pro and Val187Glyfs*13 variants (green) costained with anti-KDEL (red) in SK-N-SH cells. Cells were treated with MG132 for 2 hours before fixation as indicated. DAPI was used to stain nuclei. (**B**) REEP6.1 variants have lower levels of steady state expression than REEP6.1 WT. Graph shows mean of at least 4 replicate experiments ± 2SD, Student's t test p<0.01. Representative western blot below with β-tubulin loading control. (**C**) Cycloheximide (CHX) chase experiment shows increased turn over of variants. The graph shows the mean ± 2SD of REEP6.1 remaining after 4 hours compared to start of CHX treatment, 4 replicates. V187Gfs*13 p<0.02. Representative western blot below with β-tubulin loading control.

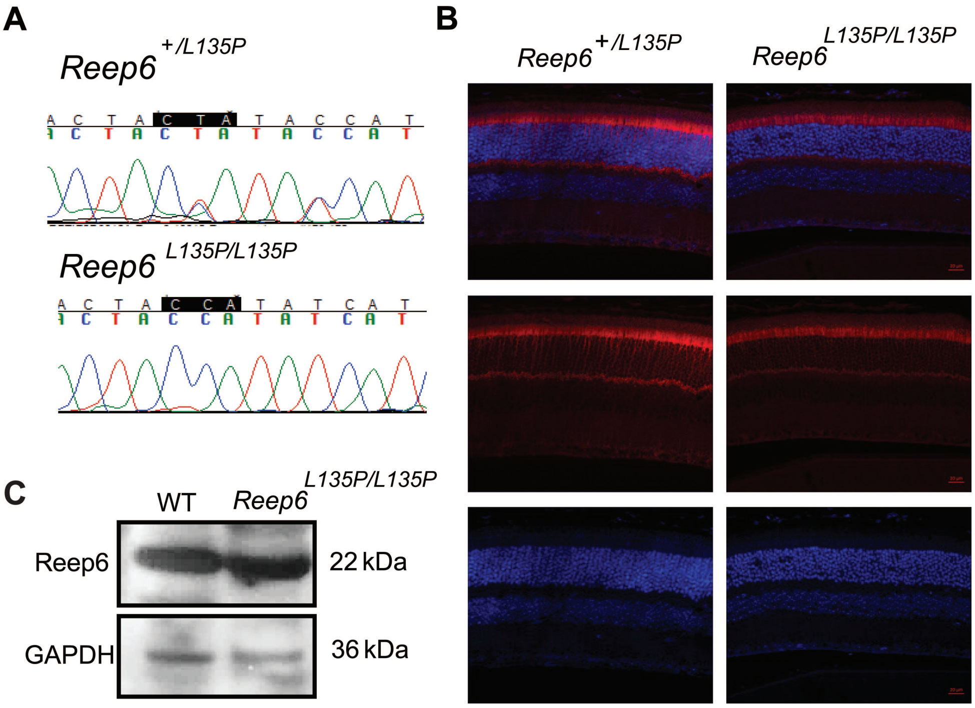

Figure S2. Genotyping of *Reep6* CRISPR/Cas9 Knock-in Mice and Reep6 Protein Expression in the Retina. (A) Chromatograms of CRISPR/Cas9 generated Reep6 heterozygous mice (*Reep6*$^{+/L135P}$) and knock-in (*Reep6*$^{L135P/L135P}$) mice. (**B**) Localization of Reep6 in *Reep6* knock-in mutant retina is unchanged compared to its localization in the *Reep6* heterozygous control retina. Scale bar: 20 µm (**C**) Western blot of retinal lysates from *Reep6* WT and *Reep6* knock-in shows that Reep6 protein levels are similar. GAPDH was used as loading control.

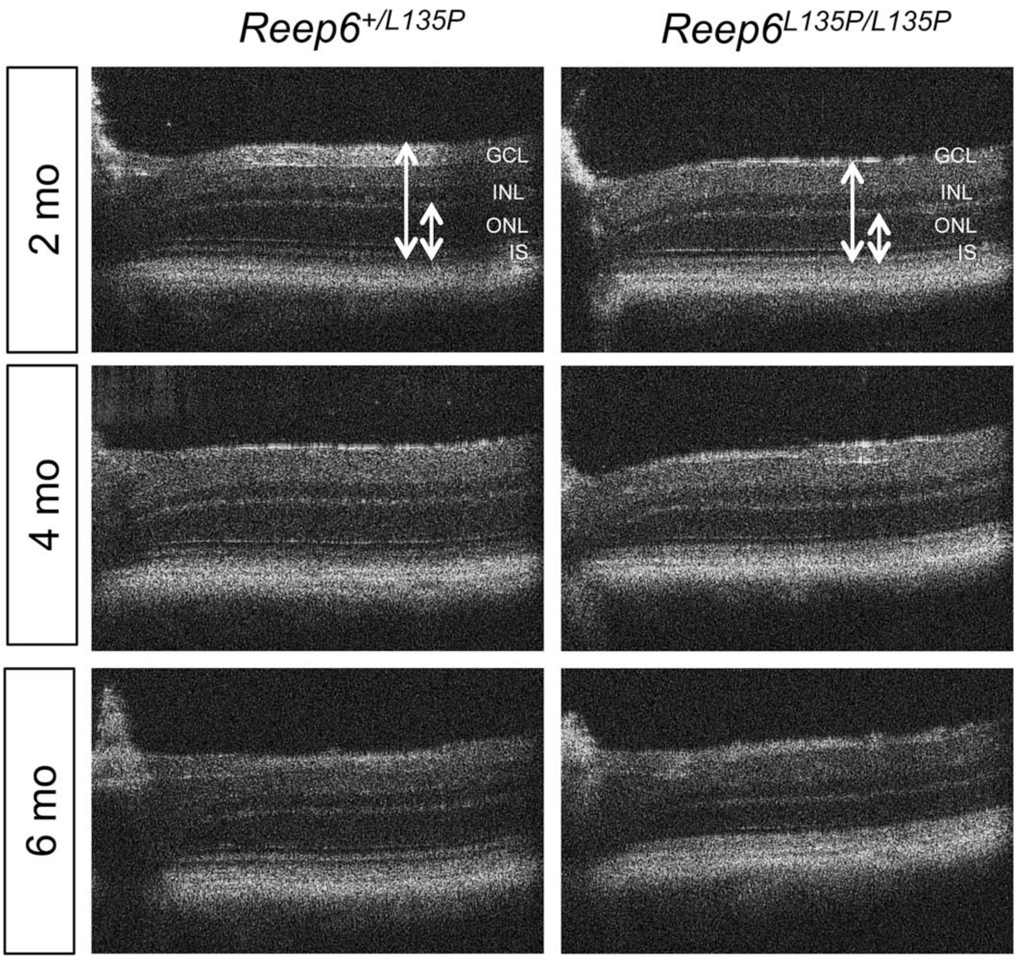

Figure S3. **SD-OCT analysis of *Reep6*$^{L135P/L135P}$ mice**. SD-OCT imaging showed thinning of the overall retina progressively (2 months to 6 months) and thinning of the ONL (arrow) in *Reep6*$^{L135P/L135P}$ mice retinas compared to littermate controls.

Chr	Coordinate	Ref	Obs	Function	Gene	Reason for exclusion	Consequence
13	32907359	A	C	nonsynonymous	BRCA2	Disease association	ENST00000544455.5: exon10:c.1744A>C, p.Thr582Pro
13	32912577	A	C	nonsynonymous	BRCA2		ENST00000544455.5: exon11:c.4085A>C, p.His1362Pro
11	67190894	C	T	stopgain	CARNS1		ENST00000445895.2: exon10:c.1675C>T, p.Gln559*
11	67191881	C	T	nonsynonymous	CARNS1	Benign prediction	ENST00000445895.2: exon10:c.2662C>T, p.Arg888Cys
20	62195499	C	T	nonsynonymous	HELZ2	LoF frequency in ExAC	ENST00000467148.1: exon9:c.4676G>A, p.Arg1559His
20	62198985	G	A	nonsynonymous	HELZ2		ENST00000427522.6 exon1:c.19C>T, p.Arg7Cys
3	195508451	G	T	nonsynonymous	MUC4	variants in cis	ENST00000463781.7: exon2:c.10000C>A, p.Pro3334Thr
3	195508453	C	T	nonsynonymous	MUC4		ENST00000463781.7: exon2:c.9998G>A, p.Ser3333Asn
3	195508454	T	C	nonsynonymous	MUC4		ENST00000463781.7: exon2:c.9997A>G, p.Ser3333Gly
19	1496339	T	C	nonsynonymous	REEP6	NA	ENST00000395484: exon4:c.404T>C, p.Leu135Pro
19	1496379	CG	C	frameshift	REEP6		ENST00000395484: exon4:c.448del, p.Ala150Profs*2
12	123957164	T	G	nonsynonymous	RILPL1	variants in cis	ENST00000376874.8: exon7:c.1133A>C, p.Glu378Ala
12	123957165	C	A	stopgain	RILPL1		ENST00000376874.8: exon7:c.1132G>T, p.Glu378*
10	99145008	A	T	nonsynonymous	RRP12		ENST00000370992.8: exon10:c.1123T>A, p.Tyr375Asn
10	99160207	G	C	nonsynonymous	RRP12	benign prediction	ENST00000370992.8: exon2:c.224C>G, p.Pro75Arg
11	19955532	C	T	nonsynonymous	NAV2	benign prediction	ENST00000528204.1: exon8:c.1811C>T, p.Ala604Val
11	19901474	Aagc	A	nonframeshift	NAV2		ENST00000528204.1: exon5:c.572_574del, p.191_192del

Table S1. Double heterozygous variants identified in individual A-II:1

Chr	Coordinate	Ref	Obs	Function	Gene	Consequence
1	27177711	A	G	nonsynonymous	ZDHHC18	ENST00000374142: exon6:c.925A>G, p.Thr309Ala
1	27238472	C	T	nonsynonymous	NR0B2	ENST00000254227: exon2:c.638G>A, p.Arg213His
1	152327526	T	A	nonsynonymous	FLG2	ENST00000388718: exon3:c.2736A>T, p.Gln912His
1	237872230	A	G	nonsynonymous	RYR2	ENST00000366574: exon69:c.9974A>G, p.Lys3325Arg
2	238736077	C	A	nonsynonymous	RBM44	ENST00000316997: exon12:c.2534C>A, p.Ala845Asp
2	241569895	C	G	nonsynonymous	GPR35	ENST00000438013: exon6:c.619C>G, p.Pro207Ala
2	242181952	C	T	nonsynonymous	HDLBP	ENST00000391975: exon17:c.2092G>A, p.Asp698Asn
2	242430588	C	T	nonsynonymous	FARP2	ENST00000264042: exon23:c.2617C>T, p.Pro873Ser
2	242814588	G	T	nonsynonymous	CXXC11	ENST00000343216: exon2:c.881G>T, p.Gly294Val
5	171765866	T	A	nonsynonymous	SH3PXD2B	ENST00000311601: exon13:c.2243A>T, p.Gln748Leu
6	18258544	T	C	nonsynonymous	DEK	ENST00000397239: exon3:c.238A>G, p.Ile80Val
10	71851666	G	C	nonsynonymous	H2AFY2	ENST00000373255: exon4:c.433G>C, p.Gly145Arg
10	96106258	G	A	nonsynonymous	NOC3L	ENST00000371361: exon11:c.1313C>T, p.Thr438Ile
15	33842470	G	A	nonsynonymous	RYR3	ENST00000389232: exon10:c.925G>A, p.Ala309Thr
15	48056050	G	A	nonsynonymous	SEMA6D	ENST00000316364: exon10:c.751G>A, p.Val251Met
15	50489851	G	T	nonsynonymous	SLC27A2	ENST00000267842: exon2:c.633G>T, p.Arg211Ser
16	30518143	C	T	nonsynonymous	ITGAL	ENST00000356798: exon21:c.2474C>T, p.Pro825Leu
16	31146747	G	A	nonsynonymous	PRSS8	ENST00000317508: exon1:c.73C>T, p.Arg25Trp
16	31193983	A	G	nonsynonymous	FUS	ENST00000254108: exon3:c.188A>G, p.Asn63Ser
16	31226444	C	T	nonsynonymous	TRIM72	ENST00000322122: exon2:c.385C>T, p.Leu129Phe
16	31373496	A	C	nonsynonymous	ITGAX	ENST00000268296: exon11:c.1187A>C, p.Glu396Ala
16	46993334	ATA A	-	Splice region	DNAJA2	ENST00000317089: c.775-6_775-3del
16	72821890	G	A	nonsynonymous	ZFHX3	ENST00000268489: exon10:c.10285C>T, p.Arg3429Cys
16	81219214	G	A	nonsynonymous	PKD1L2	ENST00000337114: exon11:c.1880C>T, p.Pro627Leu
16	82033626	T	C	nonsynonymous	SDR42E1	ENST00000328945: exon3:c.272A>G, p.Asn91Ser
16	87902985	G	A	nonsynonymous	SLC7A5	ENST00000261622: exon1:c.44C>T, p.Ala15Val
16	89925741	A	T	nonsynonymous	SPIRE2	ENST00000378247: exon9:c.1441A>T, p.Arg481Trp
17	48559747	A	C	nonsynonymous	RSAD1	ENST00000258955: exon4:c.770A>C, p.Tyr257Ser
17	48560058	G	A	nonsynonymous	RSAD1	ENST00000258955: exon5:c.895G>A, p.Val299Ile
17	48614425	C	T	nonsynonymous	EPN3	ENST00000268933: exon2:c.508C>T, p.Arg170Cys
17	48618328	C	T	nonsynonymous	EPN3	ENST00000268933: exon7:c.1154C>T, p.Thr385Ile
17	48618933	A	T	nonsynonymous	EPN3	ENST00000268933:

						exon9:c.1463A>T, p.Lys488Ile
17	48619437	-	CCGCCCCCAG CTGGCCTG	nonframeshift insertion	EPN3	ENST00000268933: exon10:c.1818_1819insCCGCCCCCAGCTGGCCTG, p.Ala606delinsAlaProProProAlaGlyLeu
17	48632895	C	T	nonsynonymous	SPATA20	ENST00000006658: exon17:c.2281C>T, p.Arg761Cys
17	74155492	C	T	nonsynonymous	RNF157	ENST00000269391: exon12:c.1228G>A, p.Val410Ile
19	1496625	-	C	frameshift insertion	REEP6	Genbank_EAW69491.1: Exon5:c.557dup, p.Val187Glyfs*13

Table S2. Homozygous rare variants identified in individual B-II:8

Family; Patient ID	Age/ gender/ country of origin	Age of onset/ symptoms	BCVA, logMAR (Snellen)	Refraction	Fundus	Age and summary of ERG	Other findings
EG76; A-II:1	35/M/Asia	5 nyctalopia,<br>18 field constriction	NA	NA	Peripheral retinal atrophy vessel attenuation bony spicules	20, only photopic responses tested, severely reduced	Goldmann visual fields reduced to 30 degrees with preserved temporal islands, early posterior subcapsular cataract
GC18419; B-II:8	54/M/Sudan	20 nyctalopia, 30 reduced fields, 40 reduced central vision	R 0.3 (20/40)<br>L 0.18 (20/30)	r +2.25/-1.25 x 75 L +2.5/-2 x 20	Attenuated vessels, mid-peripheral RPE atrophy, bone spicules and atrophic patches, CME	NA	Fields to confrontation 10 degrees, Ishihara 17/17 each eye, bilateral early cataract, anosmia age 48 years
GC20453; C-II:1	44/M/Turkey	10 nyctalopia, 37 reduced central vision	R 0.3 (20/40)<br>L 0.3 (20/40)	R pseudophakic, L -1.25/-0.75 x180	Optic disc pallor, attenuated vessels, mid-peripheral RPE atrophy, bone spicules and atrophic patches	NA	Fields to confrontation less than 10 degrees, Ishihara 17/17 each eye
GC20277; D-II:5	32/M/Iran	Early childhood nyctalopia, gradual field loss since	R 1.3 (20/400)<br>L 0.9 (20/160)	BE pseudophakic when assessed	Attenuated vessels, mid-peripheral RPE atrophy, minimal pigment change	Mid 20s, undetectable ERGs (scotopic and photopic)	Fields to confrontation 10-15 degrees, Ishihara 0/17 each eye
GC15672; E-II:1	29/M/India	14 nyctalopia, late teens fields loss	R 0.3 (20/40)<br>L 0.18 (20/30)	Myope	Attenuated vessels, mid-peripheral RPE atrophy and bone spicule hyperpigmentation, CME	15, PERG normal; rod ERG undetectable; delayed and profoundly reduced cone specific responses. Severe rod-cone dystrophy.	Fields to confrontation reduced to 30 degrees, early posterior subcapsular cataract

Table S3. Clinical Findings for *REEP6* affected individuals. Ages are indicated in years, M: male, F: female. NA: not available.
