# Supplementary material for: Mutations in REEP6 Cause Autosomal-Recessive Retinitis Pigmentosa
Source: Am J Hum Genet. 2016 Nov 23;99(6):1305–15. doi: 10.1016/j.ajhg.2016.10.008 (PMC5142109; doi:10.1016/j.ajhg.2016.10.008)
Supplement: Document S2. Article plus Supplemental Data [file mmc2.pdf]

Mutations in *REEP6* Cause Autosomal-Recessive Retinitis Pigmentosa

Gavin Arno,[1,2,13] Smriti A. Agrawal,[3,4,13] Aiden Eblimit,[3,4] James Bellingham,[1] Mingchu Xu,[3,4] Feng Wang,[3,4] Christina Chakarova,[1] David A. Parfitt,[1] Amelia Lane,[1] Thomas Burgoyne,[1] Sarah Hull,[1,2] Keren J. Carss,[5,6] Alessia Fiorentino,[1] Matthew J. Hayes,[1] Peter M. Munro,[1] Ralph Nicols,[7] Nikolas Pontikos,[1] Graham E. Holder,[1,2] UKIRDC, Chinwe Asomugha,[8] F. Lucy Raymond,[5,9] Anthony T. Moore,[1,2,10] Vincent Plagnol,[11] Michel Michaelides,[1,2] Alison J. Hardcastle,[1] Yumei Li,[3,4] Catherine Cukras,[12] Andrew R. Webster,[1,2] Michael E. Cheetham,[1,*] and Rui Chen[3,4,*]

Retinitis pigmentosa (RP) is the most frequent form of inherited retinal dystrophy. RP is genetically heterogeneous and the genes identified to date encode proteins involved in a wide range of functional pathways, including photoreceptor development, phototransduction, the retinoid cycle, cilia, and outer segment development. Here we report the identification of biallelic mutations in Receptor Expression Enhancer Protein 6 (*REEP6*) in seven individuals with autosomal-recessive RP from five unrelated families. REEP6 is a member of the REEP/Yop1 family of proteins that influence the structure of the endoplasmic reticulum but is relatively unstudied. The six variants identified include three frameshift variants, two missense variants, and a genomic rearrangement that disrupts exon 1. Human 3D organoid optic cups were used to investigate *REEP6* expression and confirmed the expression of a retina-specific isoform *REEP6.1*, which is specifically affected by one of the frameshift mutations. Expression of the two missense variants (c.383C>T [p.Pro128Leu] and c.404T>C [p.Leu135Pro]) and the REEP6.1 frameshift mutant in cultured cells suggest that these changes destabilize the protein. Furthermore, CRISPR-Cas9-mediated gene editing was used to produce *Reep6* knock-in mice with the p.Leu135Pro RP-associated variant identified in one RP-affected individual. The homozygous knock-in mice mimic the clinical phenotypes of RP, including progressive photoreceptor degeneration and dysfunction of the rod photoreceptors. Therefore, our study implicates *REEP6* in retinal homeostasis and highlights a pathway previously uncharacterized in retinal dystrophy.

Introduction

Retinitis pigmentosa (RP [MIM: 268000]) is the most common inherited retinal dystrophy, affecting approximately 1 in 4,000 individuals[1] and resulting in more than 1 million visually impaired individuals worldwide. RP is genetically heterogeneous with autosomal-dominant, autosomal-recessive, and X-linked modes of inheritance and at least 58 genes associated with an autosomal-recessive form (arRP; RetNet). These genes encode proteins involved in a diverse range of functional pathways in the neural retina including photoreceptor development, phototransduction, retinoid cycle, cilia, and outer segment development and protein transport.[2,3] Mutations in any one of these many pathways leads to a remarkably similar phenotype characterized by rod photoreceptor dysfunction and degeneration and subsequent cone degeneration.[4,5] Affected individuals commonly present with nyctalopia and peripheral visual field constriction; there can be severe vision loss if the macular cones become involved. Despite the development of targeted next-generation sequencing screening strategies for identifying pathogenic variants in genes already associated with RP, an estimated 40% of cases remain without a molecular diagnosis,[6] suggesting that mutations may exist in genes not previously associated with Mendelian disease.

Here we report the identification of biallelic mutations in *Receptor Expression Enhancer Protein 6* (*REEP6* [MIM: 609346]) in individuals with arRP from five unrelated families. *REEP6* encodes a putative endoplasmic reticulum (ER) shaping factor, which is highly expressed in rod photoreceptor cells. All six alleles we have identified are predicted to be loss-of-function variants, including one allele that disrupts the rod photoreceptor-specific isoform *REEP6.1*. A knock-in mouse model of one of the identified missense variants leads to retinal degeneration and confirms that REEP6 function is important for retinal homeostasis.

[1]UCL Institute of Ophthalmology, 11-43 Bath Street, London EC1V 9EL, UK; [2]Moorfields Eye Hospital, London EC1V 2PD, UK; [3]Department of Molecular and Human Genetics, Baylor College of Medicine, Houston, TX 77030-3411, USA; [4]Human Genome Sequencing Center, Baylor College of Medicine, Houston, TX 77030-3411, USA; [5]NIHR BioResource - Rare Diseases, Cambridge University Hospitals NHS Foundation Trust, Cambridge Biomedical Campus, Cambridge CB2 0QQ, UK; [6]Department of Haematology, University of Cambridge, NHS Blood and Transplant Centre, Cambridge CB2 0PT, UK; [7]Department of Ophthalmology, Baylor College of Medicine, Houston, TX 77030-3411, USA; [8]Department of Molecular Physiology and Biophysics, Baylor College of Medicine, Houston, TX 77030-3411, USA; [9]Department of Medical Genetics, Cambridge Institute for Medical Research, University of Cambridge, Cambridge CB2 0XY, UK; [10]Ophthalmology Department, UCSF School of Medicine, Koret Vision Center, San Francisco, CA 94133-0644, USA; [11]UCL Genetics Institute, University College London, London WC1E 6BT, UK; [12]National Eye Institute, NIH, Bethesda, MD 20892, USA
[13]These authors contributed equally to this work
*Correspondence: michael.cheetham@ucl.ac.uk (M.E.C.), ruichen@bcm.edu (R.C.)
http://dx.doi.org/10.1016/j.ajhg.2016.10.008.
© 2016 The Authors. This is an open access article under the CC BY license (http://creativecommons.org/licenses/by/4.0/).

Material and Methods

Clinical Methods

The study protocol adhered to the tenets of the Declaration of Helsinki and received approval from the appropriate local ethics committees. Written, informed consent was obtained from all participants or parents of children prior to their inclusion in this study. Individual A-II:1 was diagnosed in the United States and referred by eyeGENE-approved certified eye specialists and informed consent was obtained. Clinical data and family history were obtained by contacting the referring clinician to the eyeGENE database. For the UK cohort, DNA from six affected individuals from four families (families B, C, D, E) were available for genotyping and four of these individuals underwent detailed clinical examination and retinal imaging, including 35-degree color fundus photography (Topcon Great Britain), ultra-widefield confocal scanning laser imaging with Optos (Optos plc), or Spectralis (Spectralis, Heidelberg Engineering) fundus autofluorescence (FAF) imaging and Spectralis optical coherence tomography (OCT).

Whole-Exome Sequencing

Individual A-II:1 underwent retinal capture sequencing (RCS) and whole-exome sequencing (WES) at the Human Genome Sequencing Center, Baylor College of Medicine. In brief, about 1 μg of peripheral blood mononuclear cell (PBMC)-derived genomic DNA was processed for RCS to screen for variants in known disease-causing genes as described previously.[7] For WES, the NimbleGenSeqCap EZ Hybridization and Wash kit (NimbleGenSeqCap EZ Human Exome Library v.2.0) was used according to the manufacturer's protocols. Sequencing of captured libraries was performed on Illumina HiSeq 2000 (Illumina) as 100 bp paired-end reads according to the manufacturer's protocol. Reads were mapped to hg19 human reference sequence (build GRCh37) using Burrows-Wheeler Aligner.[8] Base quality recalibration, local realignment, and variant calling were performed as previously described.[7] Variant frequencies were obtained from a series of public and internal control databases[7] as well as the Exome Aggregation Consortium (ExAC) database. Since RP is a Mendelian disorder, variants with a minor allele frequency (MAF) higher than 0.005 (for a recessive model) or 0.0001 (for a dominant model) were filtered out. After frequency-based filtering, synonymous variants were filtered out and the pathogenicity of the remaining variants were predicted using SIFT,[9] PolyPhen-2,[10] LRT,[11] MutationTaster,[12] and MutationAssessor[13] as previously described.[7] Genes with biallelic variants were considered, assuming autosomal-recessive inheritance (Table S1). Variants were ranked based on their pathogenicity score and biological plausibility.

Individual B-II:8 underwent WES as part of a large collaborative study on rare inherited retinal dystrophy (UK Inherited Retinal Dystrophy Consortium) at the University of Leeds Next Generation Sequencing Facility, UK. In brief, 200 ng of PBMC-derived genomic DNA was processed according to the Agilent SureSelect XT Library Prep protocol (Agilent Technologies) with exons being captured using the SureSelect Exome V5 Capture library. After hybridization and indexing, six samples were pooled and 100 bp paired end sequencing was performed (Illumina HiSeq 2500 sequencer). Reads were aligned to the hg19 human reference sequence (build GRCh37) using Novoalign v.2.08. Duplicate reads were marked with Picard tools MarkDuplicates. Calling was performed using GATK, creating gVCF formatted files for each sample. The individual gVCF files for the individual discussed in this study, in combination with ~4,500 clinical exomes (UCL-exomes consortium), were combined into merged VCF files for each chromosome containing on average 100 samples each. The final variant calling was performed using the GATK Genotype gVCFs module jointly for all samples (cases and controls). Variant quality scores were then re-calibrated according to GATK best practices separately for indels and SNVs. Resulting variants were annotated using ANNOVAR based on Ensembl gene and transcript definitions. Candidate variants were filtered based on function (non-synonymous, presumed loss-of-function, or splicing) and MAF (<0.005) in an in-house exome-sequencing control dataset of approximately 1,000 individuals (UCLEx), NHLBI GO Exome Sequencing Project (EVS), and 1000 Genomes phase 1 dataset, resulting in 549 rare variants. Based on family consanguinity, 36 homozygous variants (Table S2) were further manually interrogated for variant call quality, predicted pathogenicity, and biological plausibility.

Whole-Genome Sequencing

DNA from 599 unrelated affected individuals with inherited retinal disease, ascertained from the Inherited Eye Disease clinics at Moorfields Eye Hospital (MEH), London, underwent whole-genome sequencing (WGS) as part of the National Institute of Health Research (NIHR) BioResource – Rare Diseases project. In brief, PBMC-derived genomic DNA was processed using the Illumina TruSeq DNA PCR-Free Sample Preparation kit (Illumina) and sequenced using an Illumina Hiseq 2500, generating minimum coverage of 15× for ~95% of the genome. Reads were aligned to hg19 human reference sequence (build GRCh37) using Isaac aligner (Illumina). SNVs and indels were identified using Isaac variant caller. Variant examination was performed only on the SNVs and indels that met the following criteria: passed standard quality filters, predicted to alter the sequence of a protein, and had an MAF <0.01 in the 1000 Genomes database, EVS, the UK10K database, and ExAC and <0.02 in ~6,000 internal control genomes. In the first instance, likely disease-causing variants in a panel of 192 genes previously associated with inherited retinal disease were interrogated (gene list available on request).

Sanger Sequencing

In order to confirm the *REEP6* variants identified by next-generation sequencing, bi-directional direct Sanger sequencing was performed using specific oligonucleotide primers flanking the exons. Family segregation was performed in all available family members (Figure 1). 400 RP probands without a molecular diagnosis were screened for mutations in *REEP6* by bi-directional direct Sanger sequencing of all coding exons and intron/exon boundaries (primers and conditions available on request).

Human iPSC Photoreceptor Differentiation and Analyses

Control iPSCs were generated by reprogramming fibroblasts as described previously.[14] iPSCs were maintained on geltrex in mTESR-E8 media (Stem Cell Technologies). Directed differentiation of iPSCs into three-dimensional optic cups was based on the protocol by Nakano et al.[15] In brief, embryoid bodies (EBs) were generated in V-bottomed 96-well plates and differentiated for 18 days prior to transfer to non-adherent dishes. Pouches of transparent neuroepithelium were manually isolated from EBs under a dissecting microscope at day 30 and transferred to fresh

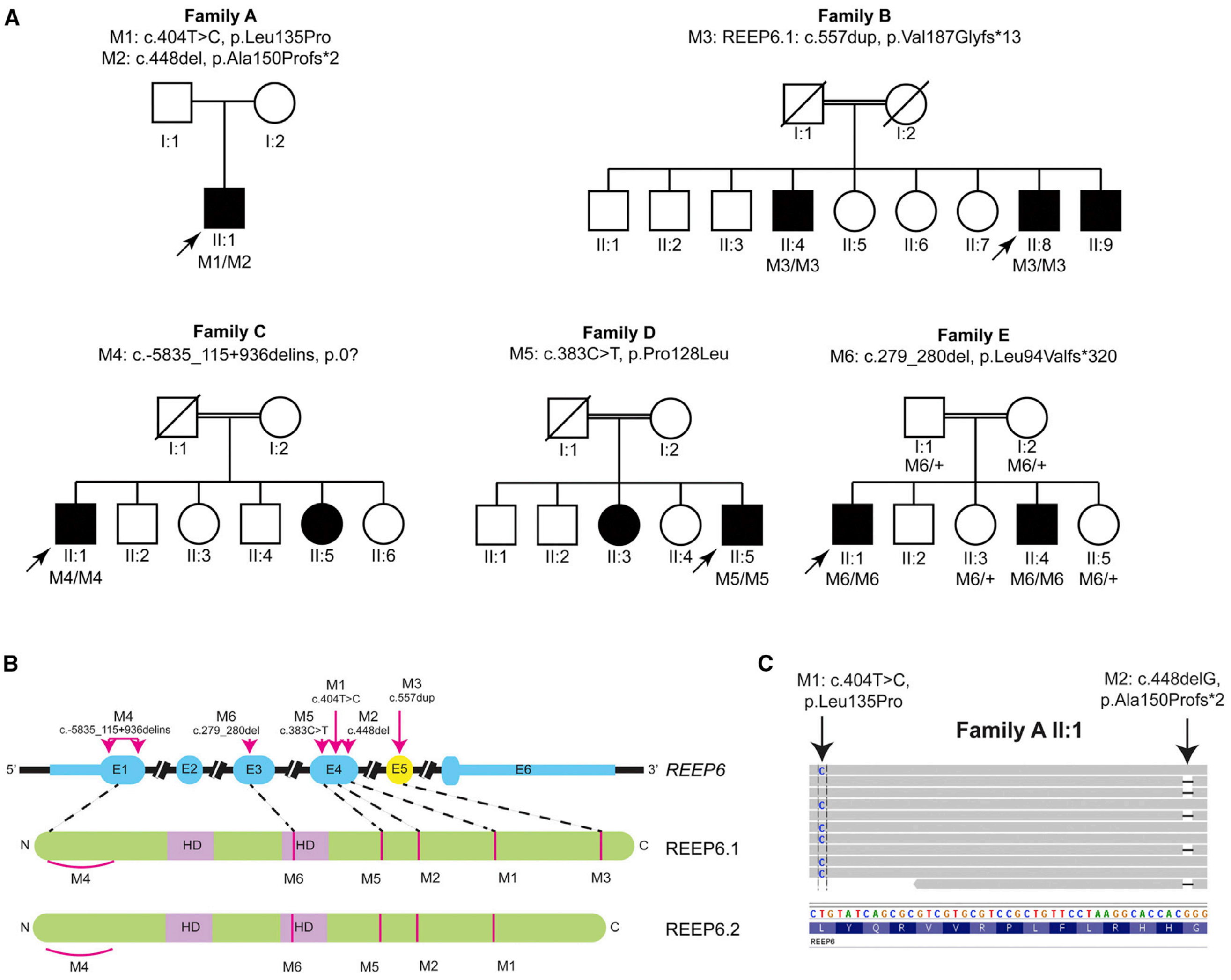

Figure 1. Autosomal-Recessive RP Families and Associated *REEP6* Variants

(A) Pedigrees of all families with RP in this study (families A to E). Proband II.1 from family A is compound heterozygous for *REEP6* variants (M1/M2). All other affected individuals were homozygous for the respective *REEP6* variants indicated (M3, M4, M5, M6). M3 c.−5835_115+936delinsAC027307.5:79083_79240inv is abbreviated to c.−5835_115+936delins.

(B) *REEP6* gene structure with nucleotide positions for M1 to M6 mapped on to exons (E1–E6). Exon 5 is present only in isoform *REEP6.1* (absent from *REEP6.2*). Protein structure of REEP6.1 and REEP6.2 with position of the corresponding mutations indicated.

(C) Sequence reads from individual A-II:1 aligned against + strand of hg19 showing heterozygous mutations in *REEP6*. Both mutations are in exon 4: c.T404C (p.Ala150Profs*2) and c.448delG (p.Leu135Pro) are in *trans*.

non-adherent dishes. These were maintained for up to 21 weeks. Optic cups were selected after morphological assessment under a light microscope at several time points during the differentiation and fixed in 4% paraformaldehyde (PFA) for 40 min at 4°C, cryo-protected by incubation overnight in 30% sucrose in phosphate buffer saline (PBS), embedded in OCT compound (Sakura Finetek), frozen, and cryosectioned (6 μm sections). Cryosectioned optic cups were incubated in blocking buffer (10% normal goat serum [NGS], 0.1% Triton X-100 in PBS) for 1 hr at room temperature before incubation with primary antibodies (mouse anti-rhodopsin 4D2 [1:500; Millipore], rabbit anti-REEP6 [1:100; Proteintech cat# 120889-1-AP], mouse anti-cone-arrestin clone 7G6 [1:100; kind gift of Peter MacLeish, Morehouse School of Medicine, Atlanta, GA, USA]) for 2 hr at room temperature. Species-specific anti-IgG Alexa Fluor 488 or 594 secondary antibodies were used as appropriate. Nuclei were visualized using DAPI (2 μg/mL) staining for all images. RNA was extracted and synthesis of cDNA was performed using Tetro cDNA synthesis kit (Bioline) for reverse transcription. GoTaq Green (Promega) was used for amplification by PCR with using standard protocols and conditions with primers *REEP6* F: 5′-TCCTGTCCTGGTTCCCTTTC-3′ and *REEP6* R: 5′-GGC TGCTTCACTTGTCCTTC-3′.

REEP6 Expression Constructs and Transient Transfection

The *REEP6* splice variant open reading frames were amplified from control D124 optic cup cDNA[16] using the primers *REEP6*_F1, 5′-GCTAGCCACCATGGACGGCCTGAGGCAGCGCGTGGAG-3′, and *REEP6*_R1stop, 5′-AATCTAGAGCGGCCGCTCACTTGTCCTT CGGCTGCGGGGTCTGGC-3′. The two observed PCR amplicons corresponded to the predicted *REEP6.1* and *REEP6.2* sizes and were excised and gel purified (QIAquick Gel Extraction Kit, QIAGEN) prior to cloning into the pSC-B-amp/kan vector

(StrataClone Blunt PCR Cloning Kit, Agilent Technologies). Clone identities (*REEP6.1*, GenBank: NM_001329556.1; *REEP6.2*, GenBank: NM_138393.2) were confirmed by Sanger sequencing (Source BioScience). Mutations were introduced by site-directed mutagenesis (Q5 Site-Directed Mutagenesis Kit, New England Biolabs) using primers and conditions specified by the NEBaseChanger software. Sequence integrity was confirmed by Sanger sequencing as before. To create expression vectors, wild-type (WT) and mutant *REEP6.1* sequences were cloned into pEYFP-N1 (Clontech) digested with *Bmt*I and *Not*I to release the EYFP sequence. WT and mutant *REEP6.1* expression plasmids were transfected into SK-N-SH (ATCC) cells using *Trans*IT-LT1 Transfection Reagent (Mirus Bio) using the manufacturers' recommended conditions, in 8-well Nunc Lab-Tek Permanox chamber slides (ThermoFisher Scientific) plated at 40,000 cells/well and 6-well plates plated at 500,000 cells/well.

Immunocytochemistry

At 20 hr after transfection, SK-N-SH neuroblastoma cells in 8-well chamber slides were treated for 2 hr with 50 μM MG-132 (in DMSO) or the vehicle DMSO. Standard protocols were used for immunocytochemistry. In brief, transfected SK-N-SH cells in chamber slides were washed with PBS and fixed for 15 min with 4% formaldehyde (in PBS) and permeabilized with 0.1% Triton X-100 (in PBS) for 5 min, and non-specific antibody binding was blocked for 1 hr using 3% BSA and 10% secondary antibody species serum in PBS. Primary antibodies, rabbit anti-REEP6 (1:200; Proteintech) and mouse anti-KDEL (1:200; Enzo Life Sciences cat# ADI-SPA-827-F), were applied for 1 hr in the blocking solution. Appropriate Alexa Fluor 488 and 594 secondary antibodies were incubated for 45 min in the blocking solution. Nuclei were labeled with DAPI (in PBS) and coverslips mounted with Fluorescence Mounting Medium (Dako). Cells were imaged on an Zeiss LSM 700 Confocal Microscope. Digital processing was undertaken with ImageJ and figures prepared in Graphic (Autodesk).

Cycloheximide Treatment

At 20 hr after transfection, SK-N-SH cells in 6-well plates were treated with 50 μM cycloheximide. Cells were harvested at T = 0, +2, and +4 hr cycloheximide exposure time points. Cells were washed twice with PBS and collected in 300 μL RIPA buffer before brief sonication. 5× sample buffer was added to aliquots prior to thermal denaturation and prior to resolution by SDS-PAGE (15%) and western blotting. Western blots were blocked in 5% Marvel (in PBS) prior to incubation with rabbit anti-REEP6 (1:2,000; Proteintech) and mouse anti-β-tubulin (1:3,000; Sigma-Aldrich, cat# T4026). Horseradish peroxidase (HRP)-conjugated secondary antibodies (1:30,000) were visualized with Pierce ECL 2 Substrate (ThermoScientific). Western blots were visualized on a Bio-Rad ChemiDoc MP Imaging System and quantitated using Image Lab 5.2.1 (Bio-Rad Laboratories). Statistical significances were calculated by Student's t test.

Generation of *Reep6* Knock-in Mice using CRISPR Targeting

Reep6$^{L135P/L135P}$ mice harboring the c.404T>C mutation identified in individual A-II:1 were generated using the CRISPR-Cas9 gene-targeting approach. All animal operations were approved by the Institutional Animal Care and Use Committee at Baylor College of Medicine. Single-guide RNA (sgRNA) target site for murine *Reep6* close to the disease variant in exon 4 was selected using CRISPR design tool (5′-AGAGTGGTCTTATGACGCGATGG-3′). Additionally, the donor template contained a silent mutation (c.408C>T) to avoid sgRNA recognition and excision. The murine *Reep6* sgRNA was cloned into pDR274, a cloning vector (Addgene) to form a T7 promoter-mediated sgRNA expression vector. BsaI digestion was performed to linearize the vector. After gel purification (QIAGEN), we used the linearized expression vector as a template to produce sgRNA using the Maxiscript T7 kit (Life Technologies) and purified with RNA Clean and Concentrator-25 (Zymo Research). RNA concentration was measured using a NanoDrop ND1000. Single-stranded donor oligonucleotides (ssODN) spanning the region were designed and contained the disease variant and an extra mutation at the PAM site to avoid degradation of the donor by Cas9. ssODN was ordered from IDT as an ultramer (5′-CTTCCTGTTATTTTGCATGACGCCCGGACC CTGGAACGGGGCATTACTACCATATCATCGCGTCATAAGACCA CTCTTTCTAAAGCACCACATGGCTCTAGACAGCGCCGCGAGC CAGCT-3′). To make cas9 mRNA, a modified pX330[17] was linearized with NotI and used as template for RNA production (mMESSAGE mMACHINE T7 Transcription Kit, Invitrogen).

For microinjections, the donor oligos (33 ng/μL) were mixed with Cas mRNA (40 ng/μL) and sgRNA (20 ng/μL) and microinjected into C57BL/6 embryos at the single-cell stage. Once the embryos reached blastocyst stage, they were transferred into the uterus of pseudopregnant females to obtain founder mice. To genotype the subsequent generations and onward of *Reep6* knock-in mice, we used a genomic PCR assay using the following primers: *Reep6*_KI_F: 5′-TCCTGTTCTGGTTCCCTTTCTA-3′ and *Reep6*_KI_M13R: 5′-CTGCTCAGGAAACAGCTATGACGGAAAAT AAATCCAGCATCCA-3′. All mice in this study were maintained under light cycles of 12-hr light and 12-hr dark.

Electroretinography

Mice were dark-adapted overnight, then anesthetized with ketamine (22 cmg/kg), xylazine (4.4 cmg/kg), and acepromazine (0.37 cmg/kg) by intraperitoneal injection. Pupils were dilated in dim red light with tropicamide (1.0%) and phenylephrine (2.5%) solutions and the cornea anesthetized with proparacaine (1.0%). Goniosoft (2.5%) was generously applied on the cornea to keep it moistened and enhance contact between the cornea and the ERG electrode. Scotopic ERG was performed at six flash intensities, −24, −14, −4, 0, and 10 dB (0.01, 0.1, 1, 2.5, 25 cd*s/m^2) on 4- to 6-month-old *Reep6*$^{L135P/L135P}$ and *Reep6*$^{+/L135P}$ mice. After completion of the scotopic ERG, mice were light-adapted to a 30 ccd/m^2 white background for 2 cmin, and photopic ERGs recordings were obtained at flash intensities of 0, 10, and 25 cdB. The LKC UTAS Visual Diagnostic System and EMWIN software (LKC Technologies) was utilized to digitize and store the recordings. ERG data was analyzed and plotted using GraphPad Prism5 software (GraphPad Software).

Spectral-Domain Optical Coherence Tomography

Total retinal thickness and thickness of the outer nuclear layer (ONL) was assessed and measured using an ultrahigh resolution Spectral Domain Ophthalmic Imaging System (Envisu R2200 SDOIS, Leica Microsystems). Retinal imaging of each eye from *Reep6 WT*, *Reep6*$^{+/L135P}$, and *Reep6*$^{L135P/L135P}$ mice was performed on mice anesthetized by intraperitoneal injection of a ketamine/xylazine cocktail (ketamine, 100 mg/kg; xylazine, 10 mg/kg) after dilating eyes with one drop 1% cyclopentolate hydrochloride

ophthalmic solution (Baush & Lomb) followed by one drop of 2.5% phenylephrine hydrochloride ophthalmic solution (Falcon Pharmaceuticals). Eyes were lubricated using Systane Ultra lubricant drops (Alcon) before dilation and GenTeal Severe lubricant gel (Alcon) after dilation. The mice were then placed in the cylindrical mouse cassette on the rodent alignment stage (RAS) in front of the optical scanning mouse retina bore. Pupils were centered using the X-, Y-, and Z-translators, and each eye was imaged along the entire axial length. To minimize the anesthesia time, all of the preparations were done in advance. The imaging of both eyes was completed within 4–5 min per mouse. Image acquisition, assessment, and processing were done using the InVivoVue 2.2 Image Acquisition Software (Leica Microsystems).

Histology and Transmission Electron Microscopy

Mice were euthanized with isoflurane followed by cervical dislocation. Eyes were enucleated from mutant and control animals and fixed overnight at 4°C in fresh Davidson's fixative.[18] Fixed eyes were processed through ethanol dehydration series (50%, 70%, 95%, 100%) for 1 hr each. Eyes were then paraffin embedded for microtome sectioning. Serial paraffin sections (7 μm) were obtained and H&E stained according a standard protocol. All H&E-stained slides were visualized using light microscopy (Zeiss Apotome) and photoreceptor nuclei were counted and quantified. For transmission electron microscopy (TEM), fresh eye cups were dissected from mice and fixed in EM fixative comprising 2.5% glutaraldehyde, 1% PFA, and 0.1 M PBS (pH 7.3) overnight at 4°C. Samples were post fixed in 1% osmium tetroxide and dehydrated in a series of graded alcohols. Conjunctiva samples were infiltrated with acetone and PolyBed 812 plastic resin and embedded in plastic molds with 100% plastic resin. Sections of 1 micron (thick) and 80–90 nm (thin) were cut (Leica Ultracut R ultramicrotome). Thick sections were stained with Touludine Blue stain and thin sections were placed on copper mesh grid stained with uranyl acetate and lead citrate. Zeiss EM902 was used for visualizing the sections and imaging was performed on AMT V602 digital camera.

Results

Biallelic *REEP6* Mutations Identified in Individuals with Retinitis Pigmentosa

Using a combination of WES, WGS, and direct Sanger sequencing, mutations in *REEP6* were identified in seven individuals with RP from five unrelated families.

A simplex RP individual (A-II:1), an Asian male from a non-consanguineous family, and individual B-II:8, one of three male siblings with RP born to consanguineous parents of African descent, were selected for WES after negative screening by targeted capture sequencing of 226 and 176 genes known to harbor pathogenic mutations associated with inherited retinal disease, respectively. WES data revealed 463 rare single-nucleotide variants (SNVs) for individuals A-II:1 and 549 rare SNVs were identified for individual B-II:8. For individual A-II:1, based on the presumed recessive inheritance, genes with two or more variants were prioritized and variants were excluded in genes if at least one of the variants was predicted to be benign or neutral by at least 4/5 of the algorithms used, or if homozygous loss-of-function (LOF) genotype was observed in the ExAC database (Table S1). Based on these filtering criteria, we eliminated six out of eight genes leaving biallelic variants in *BRCA2* (MIM: 600185) and *REEP6*. *BRCA2* is a well-characterized tumor suppressor gene, mutations in which are associated with cancers of the breast, brain, pancreas, and prostate; thus, biallelic variants identified in *BRCA2* were deemed unlikely to be associated with RP based on biological plausibility. The biallelic variants in *REEP6* remained the only candidate RP-associated variants. Thirty-six homozygous variants were identified in individual B-II:8 (Table S2). Interrogation of the variant call quality, MAF in the ExAC database, predicted protein impact, prior disease association, expression pattern, and predicted or known function of the proteins led to the prioritization of *REEP6* as the most compelling candidate variant.

Individual A-II:1 (EG76) harbored compound heterozygous variants in *REEP6*: a missense mutation (GRCh37 [hg19] chr19:g.1496339T>C, GenBank: NM_138393.1; c.404T>C [p.Leu135Pro]) and a single-nucleotide deletion also in exon 4 (GRCh37 [hg19] chr19:g.1496383del, GenBank: NM_138393.1; c.448del [p.Ala150Pfs*2]) (Figures 1A and 1B). Both variants are absent in any publicly available databases (Table 1). The close proximity of the two variants, with a distance of only 44 bp, allowed interrogation of the 100 bp paired end reads using the Integrative Genomics Viewer[19,20] and confirmation of the biallelic state (Figure 1C).

A homozygous variant in *REEP6* (GRCh37 [hg19] chr19:g.1496629dup) was identified in individual B-II:8 (GC18419; Figures 1A and 1B). This variant is absent from any databases (Table 1). Interestingly, although this is intronic in the canonical *REEP6* transcript (*REEP6.2*, GenBank: NM_138393.2, c.517+177dup), it is located within exon 5 of a retinal-specific *REEP6* transcript (*REEP6.1*, GenBank: NM_001329556.1). Importantly, *Reep6.1* appears to be the major isoform expressed in the mouse retina.[21] This variant is predicted to cause a frameshift leading to 12 novel amino acid residues followed by a premature stop codon (*REEP6.1*, c.557dup [p.Val187Glyfs*13]) that affects only the retinal-specific isoform *REEP6.1*.

Subsequent interrogation of WGS data from 599 unrelated individuals with inherited retinal disease revealed a large homozygous genomic rearrangement encompassing the first exon of *REEP6* in an RP-affected individual of Turkish descent (individual C-II:1, GC20453) in the absence of plausible causative mutations in any gene known to be associated with retinal disease or other candidate genes. Examination of split reads across the rearrangement and confirmation by direct Sanger sequencing across the breakpoints revealed a homozygous deletion of 6,886 bp (GRCh37 [hg19] chr19:g.1485434_1492319) with an insertion of an inverted 158 bp segment from the middle of the deleted region (GRCh37 [hg19] chr19:g.1489259_1489416) comprising part of an AluY repeat sequence (Figures 1A

Table 1. Summary of Affected Individual Genotypes and Demographics

ID	*REEP6* Variant	Protein Consequence	Ancestry, Age of Diagnosis, Sex	Frequency of Variant in Control (ExAC) Database	Prediction
A-II:1	c.404T>C, c.448del	p.Leu135Pro, p.Ala150Profs*2	Asian, 5, M	0/119,812	damaging no product
B-II:8	*REEP6.1*: c.557dup, c.557dupC; *REEP6.2*: c.517+177dup, c.517+177dup	REEP6.1: p.Val187Glyfs*13, p.Val187Glyfs*13	Sudanese, 20, M	0/16,014	PTC in REEP6.1
C-II:1	c.−5835_115+936delins, AC027307.5:79083_79240inv, c.−5835_115+936delins, AC027307.5:79083_79240inv	p.0?, p.0?	Turkish, 10, M	NA	no product
D-II:5	c.383C>T, c.383C>T	p.Pro128Leu, p.Pro128Leu	Iranian, early childhood, M	0/119,724	damaging
E-II:1	c.279_280del, c.279_280del	REEP6.1: p.Leu94Valfs*86, p.Leu94Valfs*86; REEP6.2: p.Leu94Valfs*320, p.Leu94Valfs*320	Asian Indian, 14, M	1/120,294	PTC, out-of-frame extension

Ages are indicated in years. Abbreviations are as follows: M, male; F, female; NA, not available; PTC, premature termination codon.

and 1B, Table 1) (GRCh37 [hg19] chr19:g.1485434_1492319delinsAC027307.5:79083_79240inv) spanning exons 1–8 of *PCSK4* (MIM: 600487, GenBank: NM_017573.3; c.−1974_1069–1308delinsAC027307.5:79083_79240) on the reverse strand and *REEP6* exon 1 on the forward strand (GenBank: NM_138393.1, c.−5835_115+936delinsAC027307.5:79083_79240inv). This previously unreported rearrangement was not present in any other individual in our in-house dataset of 4,500 WGS samples. Loss of the first coding exon and upstream sequence is predicted to abolish *REEP6* and *PCSK4* expression. There were no extra-ocular findings attributable to loss of *PCSK4*.

Additional direct Sanger sequencing of all the coding exons of *REEP6* in a panel of 400 RP-affected individuals with unknown genetic etiology revealed two additional individuals (D-II:5, GC2027, and E-II:1, GC15672), of Iranian and Indian descent, respectively, with homozygous variants in *REEP6*. A homozygous missense mutation in exon 4 (GRCh37 [hg19] chr19:g.1496318C>T, GenBank: NM_138393.1, c.383C>T [p.Pro128Leu]) was identified in individual D-II:5 (Figures 1A and 1B). This unique variant affects a conserved residue and is predicted to be pathogenic (SIFT) and possibly damaging (PolyPhen-2) by in silico analysis. A two-nucleotide deletion in exon 3 (GRCh37 [hg19] chr19:g.1495537_1495538del, GenBank: NM_138393.1, c.279_280del) leading to frameshift that extends beyond the stop codon in the canonical transcript (p.Leu94Valfs*320, *REEP6.2*) and premature termination in the alternatively spliced exon of the retinal transcript (p.Leu94Valfs*86, *REEP6.1*) was identified in individual E-II:1 (Figures 1A and 1B). This variant was found in a single allele in the ExAC dataset (1/120,294). Direct Sanger sequencing of these suspected disease-associated mutations in the probands and all available affected and unaffected family members confirmed segregation with RP.

Clinical Phenotype

One affected individual from of each of the five families was available for clinical investigation; the clinical findings are summarized in Table S3. All affected individuals presented with initial symptoms of nyctalopia with a gradual decline in vision characterized by reduced peripheral visual fields and later reduced visual acuity. Onset varied from early childhood to 20 years of age. At last review with a mean age of 35.8 years (range 20 to 54), visual acuity ranged from 0.18 log MAR (Snellen 20/30) to 1.3 log MAR (Snellen 20/400). All affected individuals had significantly constricted visual fields ranging from less than 10 degrees to 30 degrees. Affected individuals from families B, C, D, and E had posterior subcapsular cataracts (PSCs) that had required cataract surgery in two cases. Fundus examination revealed attenuated retinal vessels and mid-peripheral hypopigmentary mottling due to atrophy of the retinal pigment epithelium (RPE). Intra-retinal pigmentary migration ("bone spicules") was documented in all but individual D-II:5 (Figure 2). OCT imaging of the macula performed in all affected individuals demonstrated outer retinal atrophy with centrally preserved inner segment ellipsoid (ISe) bands. In addition, individuals B-II:8 and D-II:5 had cystoid macular edema responsive to topical carbonic anhydrase inhibitors. Fundus autofluorescence (FAF) imaging revealed parafoveal rings of increased autofluorescence with widespread mid-peripheral loss of autofluorescence, which had nummular features in three affected individuals (Figure 2). Electrophysiology testing available in individuals A-II:1, E-II:4, and D-II:5 demonstrated a severe generalized retinal dystrophy with severely reduced or undetectable responses in individuals A-II:1 and D-II:5 in their twenties and a severe rod-cone dystrophy in individual E-II:4 at age 15 years. The clinical presentations, course, and electrophysiological findings were consistent with a clinical diagnosis of RP.

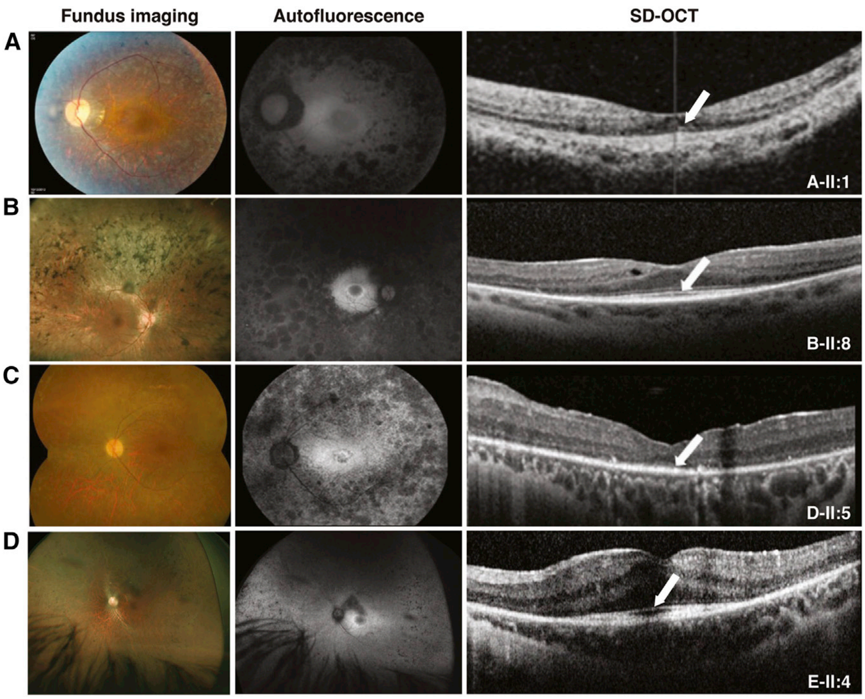

Figure 2. Clinical Findings for Individuals with Autosomal-Recessive RP Harboring *REEP6* Variants

(A) Retinal imaging of the left eye of individual II:1 at age 18 years from family A.
(B) Retinal imaging of the left eye of individual II:8 at age 32 years from family B.
(C) Right eye retinal imaging of individual II:5 at age 54 years from family D.
(D) Retinal imaging of the right eye of individual II:4 from family E.
Fundus imaging, fundus autofluorescence imaging, and optical coherence tomography (OCT) scan reveal typical retinitis pigmentosa features in all affected individuals. Color fundus photographs demonstrate severely constricted retinal arterioles, mid-peripheral retinal pigment epithelium (RPE) atrophy, retinal atrophy within and extending outward of vascular arcades, and intraretinal pigmented spicules. Fundus autofluorescence imaging demonstrates a hyperautofluorescent ring around fovea and areas of circumscribed hypoautofluorescence within and extending outward of vascular arcades. OCT demonstrates atrophy of the outer retina with small bands of retained photoreceptors (arrows) that correspond to the areas within the para-foveal rings.

Expression of *REEP6.1* in Human Photoreceptors

One of the variants identified is located in the intron (IVS4) of the canonical transcript but lies within an alternatively spliced exon in a retina-specific isoform (*REEP6.1*). This retinal isoform was identified in mice (*Reep6.1*) and includes an additional exon (exon 5) coding for 27 amino acid residues, expressed specifically in rod photoreceptors under the control of the transcription factor NRL.[21] RNA-seq data from human retinal tissue confirms the presence of the *REEP6.1* isoform in human retina[22] (annotated as GenBank: NM_001329556.1). To examine the expression of both *REEP6* isoforms in human tissue, iPSC-derived 3D organoid optic cups were utilized.[16] RT-PCR analysis over the time course of optic cup differentiation showed that the expression of the *REEP6.1* isoform correlated with the time course of photoreceptor development (Figure 3A).[16] *REEP6.1* was the predominant isoform (75%) present in mature optic cups, which is consistent with disruption of this isoform causing RP. REEP6 immunoreactivity was confined to the cell body and inner segment (IS) of developing human rod photoreceptor cells (Figure 3B) and was not detected in the rod outer segment (OS) (Figure 3B) or cone photoreceptors (Figure 3C).

Functional and Structural Consequences of *REEP6* Variants

REEP6 is a member of the REEP/Yop1 family of proteins based on protein domain homology. REEPs are proposed to influence the structure of the endoplasmic reticulum (ER), but the function of REEP6 is yet to be elucidated. REEP6 is an ER-localized protein;[23] however, the localization of the retinal isoform, REEP6.1, is not known. To test this, a WT *REEP6.1* (GenBank: NM_001329556.1) expression construct was transiently transfected in SK-N-SH neuroblastoma cells. Similar to the canonical isoform, REEP6.1 protein also localized mainly to the ER (Figure 3D). Furthermore, localization of the p.Pro128Leu, p.Leu135Pro, and p.Val187Glyfs*13 variants was also predominantly ER (Figure S1); however, inhibition of proteasome function revealed that p.Pro128Leu and p.Leu135Pro could form intracellular inclusions, suggesting a level of protein instability (Figure S1). This was confirmed by testing the steady-state expression levels, which showed that all the variants had decreased protein levels in SK-N-SH cells than the WT REEP6.1 (Figure S1) and the p.Val187Glyfs*13 showed a significantly shortened half-life (Figure S1). Furthermore, this frameshift variant is predicted to undergo nonsense-mediated decay (NMD), suggesting that the level of endogenous mRNA could also be affected. Together, these data suggest that REEP6.1 is an ER protein and that the identified variants affect protein stability.

The REEP6 p.Leu135Pro Missense Variant Is Pathogenic

To examine the function of the p.Leu135Pro variant identified in this study in vivo, a mouse knock-in model of *Reep6* p.Leu135Pro was generated using CRISPR-Cas9 technology. The $Reep6^{L135P/L135P}$ homozygous knock-in mice were viable and fertile, exhibiting no obvious morphological abnormalities upon gross examination. Immunostaining revealed no change in Reep6 localization in the $Reep6^{L135P/L135P}$ homozygous knock-in mouse retina

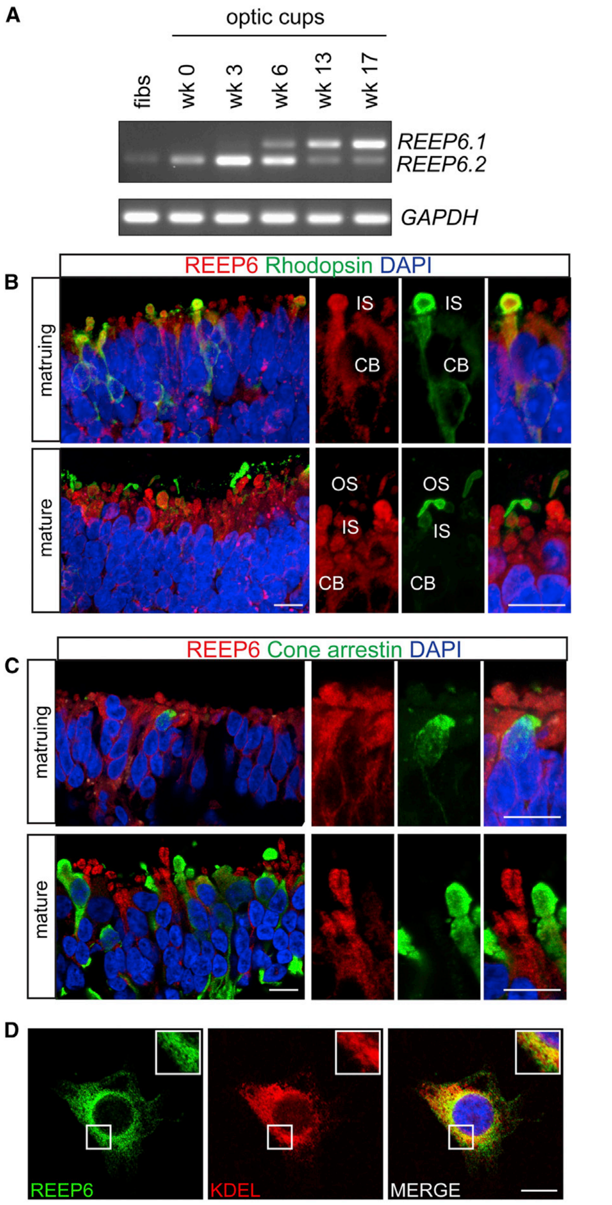

Figure 3. Expression and Localization of *REEP6* in Human iPSC Photoreceptors
(A) Reverse-transcription PCR analysis of *REEP6.1* and *6.2* isoforms in control adult fibroblasts (fibs) and control BJ optic cups during different weeks (wk) of photoreceptor differentiation. *GAPDH* used a loading control.
(B) Maturing rhodopsin-positive rod cells (green) co-express REEP6 (red) in the IS and cell body (CB). At a later stage of development, mature rods develop rhodopsin-laden outer segments (green) in which REEP6 staining (red) is not detectable despite its continued expression in the IS and CB.
(C) REEP6 (red) is not detectable in maturing cones, expressing cone arrestin (green). Mature cones (green) remain REEP6 (red) negative at this stage of development. Nuclei are counter-stained with DAPI (blue).
(D) ER localization of REEP6.1. REEP6.1 WT (green) was expressed in SK-N-SH cells, stained with anti-REEP6, and counterstained with anti-KDEL (red).
Scale bars represent 10 μm.

compared to the control retina. Additionally, western blot revealed that Reep6 protein levels were similar in *Reep6* WT and KI retinae (Figure S2). Histological analysis of the *Reep6*$^{L135P/L135P}$ retina revealed subtle changes in the overall retinal thickness and retinal morphology compared to WT or *Reep6*$^{+/L135P}$ at 4 months (Figure 4A); however, by 6 months, *Reep6*$^{L135P/L135P}$ mutant mice exhibited significant thinning of the ONL and reduced rows of nuclei when compared to *Reep6*$^{+/L135P}$ littermate controls (Figure 4B). Furthermore, retinal live imaging by SD-OCT in 2-, 4-, and 6-month-old mice was consistent with the changes observed histologically (Figure S3). To examine the defects at the ultrastructural level, transmission EM (TEM) on P20 and 6-month-old *Reep6*$^{L135P/L135P}$ mutant and *Reep6*$^{+/L135P}$ control mice was performed (Figure 4B). Although the overall retinal layer organization appeared normal in the *Reep6*$^{L135P/L135P}$ mutant retina at P20, severe alterations in the IS and OS in *Reep6*$^{L135P/L135P}$ mutant retina were evident compared to *Reep6*$^{+/L135P}$ control retina by 6 months of age (Figure 4). Electrophysiology was performed on *Reep6*$^{L135P/L135P}$ mice and *Reep6*$^{+/L135P}$ controls at 4 and 6 months. Photopic ERG recordings of 4-month-old *Reep6*$^{L135P/L135P}$ mutant mice showed normal cone activity compared to *Reep6*$^{+/L135P}$ control mice; however, the full-field scotopic ERGs showed both a- and b-wave reduction in *Reep6*$^{L135P/L135P}$ mice compared to the heterozygous littermate controls (Figure 4C) consistent with rod photoreceptor dysfunction, and this was reduced further at 6 months (data not shown). Collectively, these data demonstrate that the p.Leu135Pro missense variant is indeed pathogenic and mutation in *Reep6* causes progressive loss of rod photoreceptor function and survival in mice.

Discussion

We report biallelic mutations of *REEP6* in seven affected individuals from five families with retinal dystrophy. Mutations were not restricted to specific exons or domains of *REEP6* and include a 7 kb genomic rearrangement comprising deletion of the first exon and 5′ UTR that would abolish gene transcription, three frameshift mutations, two missense mutations, and a frameshift mutation in the rod photoreceptor-specific exon of the retinal isoform that is not expected to affect the canonical transcript. Mutations in *REEP6* have not been implicated in any human disease previously, and these data show that mutations in *REEP6* can cause inherited retinal dystrophy.

REEP6 is a member of the REEP family of proteins that are related to the Deleted in Polyposis 1 (DP1)/Yop1p family identified in yeast. Six human REEP transmembrane proteins have been annotated that were initially suggested to have a role in enhancing the expression of cell surface olfactory receptors, G protein coupled receptors (GPCR), and taste receptors.[24] REEP6 can interact with select vesicle cargo proteins, such as α2C adrenergic receptor proteins,

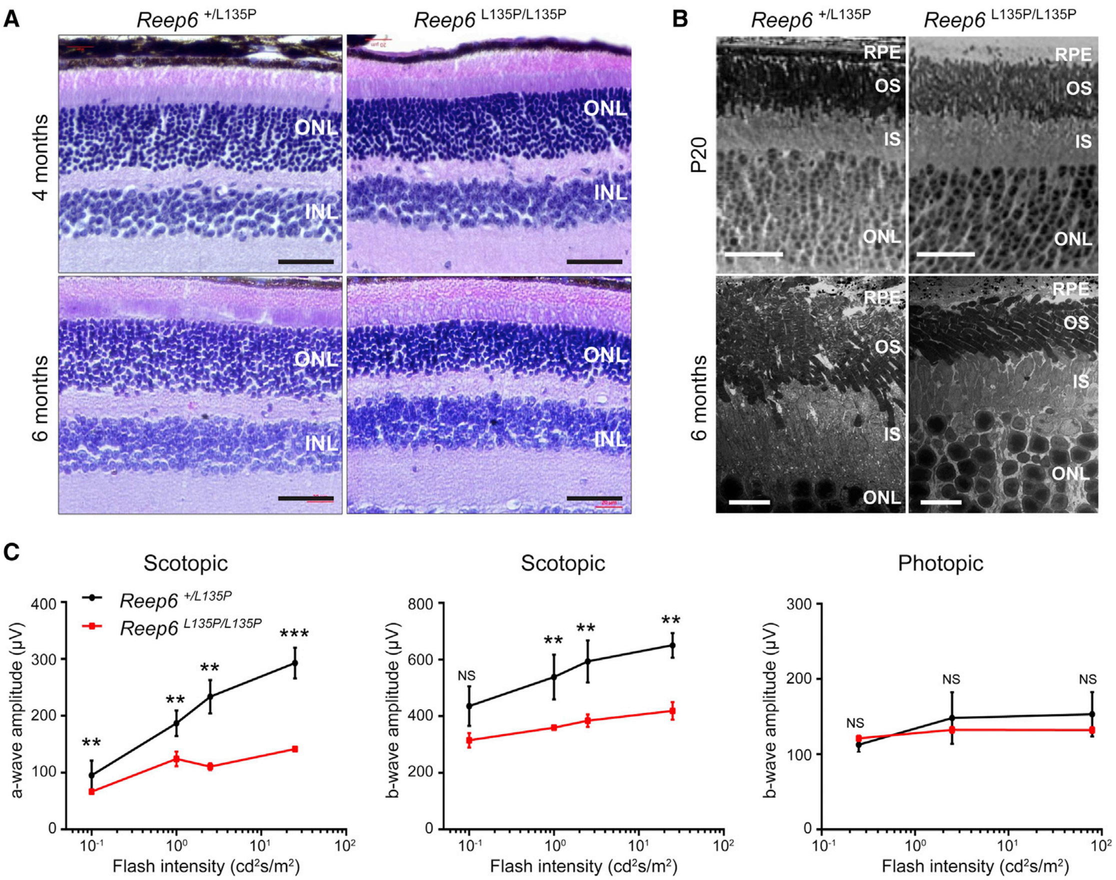

Figure 4. *Reep6*$^{L135P/L135P}$ Mutant Mice Exhibit Photoreceptor Degeneration

(A) Histological analysis of retinal sections from *Reep6*$^{+/L135P}$ (left) and *Reep6*$^{L135P/L135P}$ (right) mice was performed at 4 months and 6 months of age. *Reep6*$^{+/L135P}$ retina shows normal layered organization compared to the *Reep6*$^{L135P/L135P}$ retina that shows moderate thinning of the outer nuclear layer (ONL) at 4 months and marked thinning at 6 months of age. "INL" indicates inner nuclear layer. Scale bars represent 40 μm.

(B) TEM images of P20 and 6-month-old retina from *Reep6*$^{+/L135P}$ control retina and *Reep6*$^{L135P/L135P}$ mutant retina shows thinning of ONL, outer segment (OS), and inner segment (IS) at 6 months of age indicative of progressive retinal degeneration. Scale bars represent 2 μm (top) and 10 μm (bottom).

(C) Quantitative evaluation of scotopic a-wave and b-wave and photopic b-wave amplitude data for 4-month-old *Reep6*$^{+/L135P}$ control retina and *Reep6*$^{L135P/L135P}$ mutant mice.

NS indicates not significant, **significant at $p < 0.01$, ***significant at $p < 0.001$.

and modulate their intracellular processing by affecting the ER cargo capacity to enhance their expression at the cell surface.[23] Therefore, REEP6 could play a similar role in enhancing photoreceptor GPCR traffic and this could be probed in future studies using the knock-in mouse model.

REEPs and Yop1p are ER-resident proteins that act as membrane-shaping adaptor proteins to regulate ER membrane structure.[23,25–28] Mutations in *REEP1* (MIM: 609139) and *REEP2* (MIM: 609347) cause autosomal-dominant hereditary spastic paraplegia (MIM: 610250) and distal hereditary motor neuropathy (MIM: 614751).[29–31] Recent studies have shown that REEP1 is important in neurite development and establishing the structure of the peripheral ER[32] and in facilitating ER-mitochondria interactions.[33] Therefore, it will be relevant to study how loss of REEP6 function affects the photoreceptor ER and mitochondria. The photoreceptor ER is highly specialized to produce many of the proteins and lipids that constitute the light-sensitive OS. Furthermore, photoreceptor mitochondria are concentrated at the tip of the IS near the base of the OS; it is possible that REEP6 has a role in specializing the photoreceptor ER and/or mitochondria.

RNA-seq in mice led to the discovery of an isoform of *Reep6*, *Reep6.1*, expressed specifically in rod photoreceptors.[21] In this study we show that *REEP6.1* is the predominant isoform in developing human photoreceptors and, indeed, *REEP6* expression appears to be limited to rod photoreceptors in human 3D optic cups. The rod-specific expression pattern is consistent with the rod:cone dystrophy observed in affected individuals and the p.Leu135Pro homozygous knock-in mice. We identified a homozygous single-nucleotide duplication within intron 4 of the canonical transcript of *REEP6* that leads to a frameshift and premature termination of the *REEP6.1* transcript (c.557dupC [p.Val187Glyfs*13]) in an affected individual.

This finding further suggests that *REEP6.1* is an important transcript within rod photoreceptors and that disruption of its function alone can lead to retinal dystrophy.

The C-terminal regions of the REEP family are divergent, suggesting a specific role for this domain of the protein. Furthermore, mutations in *REEP1* often result in loss of the C-terminal region and loss of the 14-3-3 binding domain.[29,33] This domain may be important for intracellular signaling and such mutations in *REEP1* may disrupt neuronal signaling in this way without affecting the ER.[23,34] REEP6 lacks the 14-3-3 binding domain found in REEP1-4, and the REEP6.1 isoform has an additional C-terminal helix. This domain could mediate specific interactions in rod photoreceptors, potentially binding specific interacting proteins or mediating interactions between the ER and mitochondria.

Collectively, in addition to identifying variants in *REEP6* in individuals with RP, these findings highlight the utility of CRISPR-Cas9 gene editing to model human diseases. The p.Leu135Pro homozygous knock-in mice effectively replicate the RP phenotype and confirm that this variant is dysfunctional and that REEP6 is required to maintain retinal function and survival. Future studies will examine the role of REEP6 in retinal biology and understanding its involvement in maintenance of photoreceptor function and survival.

Accession Numbers

The accession numbers for the *REEP6.1* and *REEP6.2* coding sequences reported in this paper are GenBank: NM_001329556.1 and NM_138393.2.

Supplemental Data

Supplemental Data include three figures and three tables and can be found with this article online at http://dx.doi.org/10.1016/j.ajhg.2016.10.008.

Consortia

UKIRDC (UK Inherited Retinal Dystrophy Consortium) members include Graeme Black, Georgina Hall, Stuart Ingram, Rachel Gillespie, Forbes Manson, Panagiotis Sergouniotis, Chris Inglehearn, Carmel Toomes, Manir Ali, Martin McKibbin, James Poulter, Kamron Khan, Emma Lord, Andrea Nemeth, Susan Downes, Jing Yu, Stefano Lise, Gavin Arno, Alessia Fiorentino, Nikos Ponitkos, Vincent Plagnol, Michel Michaelides, Alison J. Hardcastle, Michael E. Cheetham, Andrew R. Webster, and Veronica van Heyningen.

Acknowledgments

We thank all the individuals and their family members for participating in this study. We also thank the Integrated Microscopy Core at Baylor College of Medicine for OCT experiments. Additionally, we thank Dr. Anand Swaroop for providing the Reep6 antibody and Dr. Peter MacLeish for providing the 7G6 antibody. We would also like to thank the National Ophthalmic Disease Genotyping and Phenotyping Network (eyeGENE) and Amy Turiff, the NEI coordinator, the UK Inherited Retinal Disease Consortium (UKIRDC), and the NIHR BioResource – Rare Diseases project (BRIDGE-SPEED). This project was funded by the Foundation Fighting Blindness (BR-GE-0613-0618-BCM) and National Eye Institute (R01EY022356, R01EY020540) to R.C. Sequencing effort is also supported by the NEI vision core grant (P30EY002520). The UKIRDC is funded by RP Fighting Blindness and Fight for Sight. This project was also supported by The Wellcome Trust (092621 to M.E.C.), The National Institute for Health Research (UK), Biomedical Research Centre at Moorfields Eye Hospital and UCL Institute of Ophthalmology, Fight for Sight UK, Moorfields Eye Hospital Special Trustees, and Foundation Fighting Blindness - USA.

Received: September 6, 2016
Accepted: October 13, 2016
Published: November 23, 2016

Web Resources

1000 Genomes, http://www.internationalgenome.org/
dbSNP, http://www.ncbi.nlm.nih.gov/projects/SNP/
ExAC Browser, http://exac.broadinstitute.org/
GenBank, http://www.ncbi.nlm.nih.gov/genbank/
Mutation Assessor, http://mutationassessor.org/
MutationTaster, http://www.mutationtaster.org/
NCBI Genome, http://www.ncbi.nlm.nih.gov/genome/
NCBI Nucleotide, http://www.ncbi.nlm.nih.gov/nuccore/
NEBaseChanger, http://nebasechanger.neb.com
NHLBI Exome Sequencing Project (ESP) Exome Variant Server, http://evs.gs.washington.edu/EVS/
OMIM, http://www.omim.org/
Optimized CRISPR Design, http://crispr.mit.edu/
PolyPhen-2, http://genetics.bwh.harvard.edu/pph2/
RetNet – Retinal Information Network, https://sph.uth.edu/retnet/home.htm
SIFT, http://sift.bii.a-star.edu.sg/
UCSC Genome Browser, http://genome.ucsc.edu

References

1. Ferrari, S., Di Iorio, E., Barbaro, V., Ponzin, D., Sorrentino, F.S., and Parmeggiani, F. (2011). Retinitis pigmentosa: genes and disease mechanisms. Curr. Genomics *12*, 238–249.
2. Daiger, S.P., Bowne, S.J., and Sullivan, L.S. (2007). Perspective on genes and mutations causing retinitis pigmentosa. Arch. Ophthalmol. *125*, 151–158.
3. Nash, B.M., Wright, D.C., Grigg, J.R., Bennetts, B., and Jamieson, R.V. (2015). Retinal dystrophies, genomic applications in diagnosis and prospects for therapy. Transl. Pediatr. *4*, 139–163.
4. Berson, E.L. (1993). Retinitis pigmentosa. The Friedenwald Lecture. Invest. Ophthalmol. Vis. Sci. *34*, 1659–1676.
5. Rattner, A., Sun, H., and Nathans, J. (1999). Molecular genetics of human retinal disease. Annu. Rev. Genet. *33*, 89–131.
6. Ellingford, J.M., Barton, S., Bhaskar, S., O'Sullivan, J., Williams, S.G., Lamb, J.A., Panda, B., Sergouniotis, P.I., Gillespie, R.L., Daiger, S.P., et al. (2016). Molecular findings from 537 individuals with inherited retinal disease. J. Med. Genet. *53*, 761–767, jmedgenet-2016-103837.
7. Wang, F., Wang, H., Tuan, H.-F., Nguyen, D.H., Sun, V., Keser, V., Bowne, S.J., Sullivan, L.S., Luo, H., Zhao, L., et al. (2014).

Next generation sequencing-based molecular diagnosis of retinitis pigmentosa: identification of a novel genotype-phenotype correlation and clinical refinements. Hum. Genet. *133*, 331–345.

8. Li, H., and Durbin, R. (2009). Fast and accurate short read alignment with Burrows-Wheeler transform. Bioinformatics *25*, 1754–1760.
9. Kumar, P., Henikoff, S., and Ng, P.C. (2009). Predicting the effects of coding non-synonymous variants on protein function using the SIFT algorithm. Nat. Protoc. *4*, 1073–1081.
10. Adzhubei, I.A., Schmidt, S., Peshkin, L., Ramensky, V.E., Gerasimova, A., Bork, P., Kondrashov, A.S., and Sunyaev, S.R. (2010). A method and server for predicting damaging missense mutations. Nat. Methods *7*, 248–249.
11. Chun, S., and Fay, J.C. (2009). Identification of deleterious mutations within three human genomes. Genome Res. *19*, 1553–1561.
12. Schwarz, J.M., Rödelsperger, C., Schuelke, M., and Seelow, D. (2010). MutationTaster evaluates disease-causing potential of sequence alterations. Nat. Methods *7*, 575–576.
13. Reva, B., Antipin, Y., and Sander, C. (2011). Predicting the functional impact of protein mutations: application to cancer genomics. Nucleic Acids Res. *39*, e118.
14. Okita, K., Matsumura, Y., Sato, Y., Okada, A., Morizane, A., Okamoto, S., Hong, H., Nakagawa, M., Tanabe, K., Tezuka, K., et al. (2011). A more efficient method to generate integration-free human iPS cells. Nat. Methods *8*, 409–412.
15. Nakano, T., Ando, S., Takata, N., Kawada, M., Muguruma, K., Sekiguchi, K., Saito, K., Yonemura, S., Eiraku, M., and Sasai, Y. (2012). Self-formation of optic cups and storable stratified neural retina from human ESCs. Cell Stem Cell *10*, 771–785.
16. Parfitt, D.A., Lane, A., Ramsden, C.M., Carr, A.-J.F., Munro, P.M., Jovanovic, K., Schwarz, N., Kanuga, N., Muthiah, M.N., Hull, S., et al. (2016). Identification and correction of mechanisms underlying inherited blindness in human iPSC-derived optic cups. Cell Stem Cell *18*, 769–781.
17. Zhong, H., Chen, Y., Li, Y., Chen, R., and Mardon, G. (2015). CRISPR-engineered mosaicism rapidly reveals that loss of Kcnj13 function in mice mimics human disease phenotypes. Sci. Rep. *5*, 8366.
18. Latendresse, J.R., Warbrittion, A.R., Jonassen, H., and Creasy, D.M. (2002). Fixation of testes and eyes using a modified Davidson's fluid: comparison with Bouin's fluid and conventional Davidson's fluid. Toxicol. Pathol. *30*, 524–533.
19. Robinson, J.T., Thorvaldsdóttir, H., Winckler, W., Guttman, M., Lander, E.S., Getz, G., and Mesirov, J.P. (2011). Integrative genomics viewer. Nat. Biotechnol. *29*, 24–26.
20. Thorvaldsdóttir, H., Robinson, J.T., and Mesirov, J.P. (2013). Integrative Genomics Viewer (IGV): high-performance genomics data visualization and exploration. Brief. Bioinform. *14*, 178–192.
21. Hao, H., Veleri, S., Sun, B., Kim, D.S., Keeley, P.W., Kim, J.-W., Yang, H.-J., Yadav, S.P., Manjunath, S.H., Sood, R., et al. (2014). Regulation of a novel isoform of Receptor Expression Enhancing Protein REEP6 in rod photoreceptors by bZIP transcription factor NRL. Hum. Mol. Genet. *23*, 4260–4271.
22. Farkas, M.H., Grant, G.R., White, J.A., Sousa, M.E., Consugar, M.B., and Pierce, E.A. (2013). Transcriptome analyses of the human retina identify unprecedented transcript diversity and 3.5 Mb of novel transcribed sequence via significant alternative splicing and novel genes. BMC Genomics *14*, 486.
23. Björk, S., Hurt, C.M., Ho, V.K., and Angelotti, T. (2013). REEPs are membrane shaping adapter proteins that modulate specific g protein-coupled receptor trafficking by affecting ER cargo capacity. PLoS ONE *8*, e76366.
24. Saito, H., Kubota, M., Roberts, R.W., Chi, Q., and Matsunami, H. (2004). RTP family members induce functional expression of mammalian odorant receptors. Cell *119*, 679–691.
25. Voeltz, G.K., Prinz, W.A., Shibata, Y., Rist, J.M., and Rapoport, T.A. (2006). A class of membrane proteins shaping the tubular endoplasmic reticulum. Cell *124*, 573–586.
26. Hu, J., Shibata, Y., Voss, C., Shemesh, T., Li, Z., Coughlin, M., Kozlov, M.M., Rapoport, T.A., and Prinz, W.A. (2008). Membrane proteins of the endoplasmic reticulum induce high-curvature tubules. Science *319*, 1247–1250.
27. Shibata, Y., Shemesh, T., Prinz, W.A., Palazzo, A.F., Kozlov, M.M., and Rapoport, T.A. (2010). Mechanisms determining the morphology of the peripheral ER. Cell *143*, 774–788.
28. Park, S.H., Zhu, P.-P., Parker, R.L., and Blackstone, C. (2010). Hereditary spastic paraplegia proteins REEP1, spastin, and atlastin-1 coordinate microtubule interactions with the tubular ER network. J. Clin. Invest. *120*, 1097–1110.
29. Züchner, S., Wang, G., Tran-Viet, K.-N., Nance, M.A., Gaskell, P.C., Vance, J.M., Ashley-Koch, A.E., and Pericak-Vance, M.A. (2006). Mutations in the novel mitochondrial protein REEP1 cause hereditary spastic paraplegia type 31. Am. J. Hum. Genet. *79*, 365–369.
30. Beetz, C., Pieber, T.R., Hertel, N., Schabhüttl, M., Fischer, C., Trajanoski, S., Graf, E., Keiner, S., Kurth, I., Wieland, T., et al. (2012). Exome sequencing identifies a REEP1 mutation involved in distal hereditary motor neuropathy type V. Am. J. Hum. Genet. *91*, 139–145.
31. Esteves, T., Durr, A., Mundwiller, E., Loureiro, J.L., Boutry, M., Gonzalez, M.A., Gauthier, J., El-Hachimi, K.H., Depienne, C., Muriel, M.P., et al. (2014). Loss of association of REEP2 with membranes leads to hereditary spastic paraplegia. Am J Hum Genet. *94*, 268–277.
32. Beetz, C., Koch, N., Khundadze, M., Zimmer, G., Nietzsche, S., Hertel, N., Huebner, A.-K., Mumtaz, R., Schweizer, M., Dirren, E., et al. (2013). A spastic paraplegia mouse model reveals REEP1-dependent ER shaping. J. Clin. Invest. *123*, 4273–4282.
33. Lim, Y., Cho, I.-T., Schoel, L.J., Cho, G., and Golden, J.A. (2015). Hereditary spastic paraplegia-linked REEP1 modulates endoplasmic reticulum/mitochondria contacts. Ann. Neurol. *78*, 679–696.
34. Tinti, M., Johnson, C., Toth, R., Ferrier, D.E.K., and Mackintosh, C. (2012). Evolution of signal multiplexing by 14-3-3-binding 2R-ohnologue protein families in the vertebrates. Open Biol. *2*, 120103.

The American Journal of Human Genetics, Volume *99*

Supplemental Data

Mutations in *REEP6* Cause

Autosomal-Recessive Retinitis Pigmentosa

Gavin Arno, Smriti A. Agrawal, Aiden Eblimit, James Bellingham, Mingchu Xu, Feng Wang, Christina Chakarova, David A. Parfitt, Amelia Lane, Thomas Burgoyne, Sarah Hull, Keren J. Carss, Alessia Fiorentino, Matthew J. Hayes, Peter M. Munro, Ralph Nicols, Nikolas Pontikos, Graham E. Holder, UKIRDC, Chinwe Asomugha, F. Lucy Raymond, Anthony T. Moore, Vincent Plagnol, Michel Michaelides, Alison J. Hardcastle, Yumei Li, Catherine Cukras, Andrew R. Webster, Michael E. Cheetham, and Rui Chen

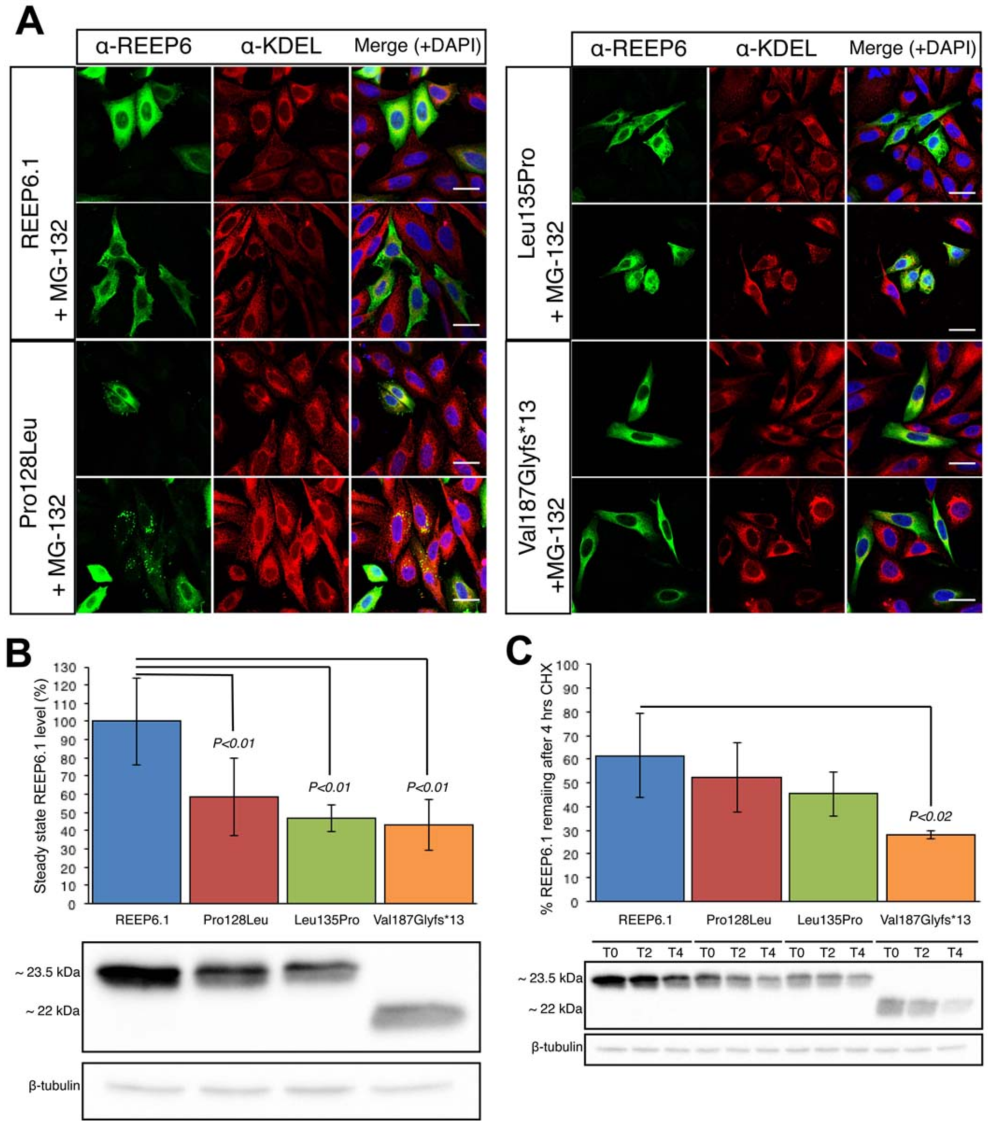

Figure S1. Variants in REEP6.1 affect protein stability. (A) The expression of REEP6.1 WT and the Pro128Leu, Leu135Pro and Val187Glyfs*13 variants (green) costained with anti-KDEL (red) in SK-N-SH cells. Cells were treated with MG132 for 2 hours before fixation as indicated. DAPI was used to stain nuclei. (**B**) REEP6.1 variants have lower levels of steady state expression than REEP6.1 WT. Graph shows mean of at least 4 replicate experiments ± 2SD, Student's t test p<0.01. Representative western blot below with β-tubulin loading control. (**C**) Cycloheximide (CHX) chase experiment shows increased turn over of variants. The graph shows the mean ± 2SD of REEP6.1 remaining after 4 hours compared to start of CHX treatment, 4 replicates. V187Gfs*13 p<0.02. Representative western blot below with β-tubulin loading control.

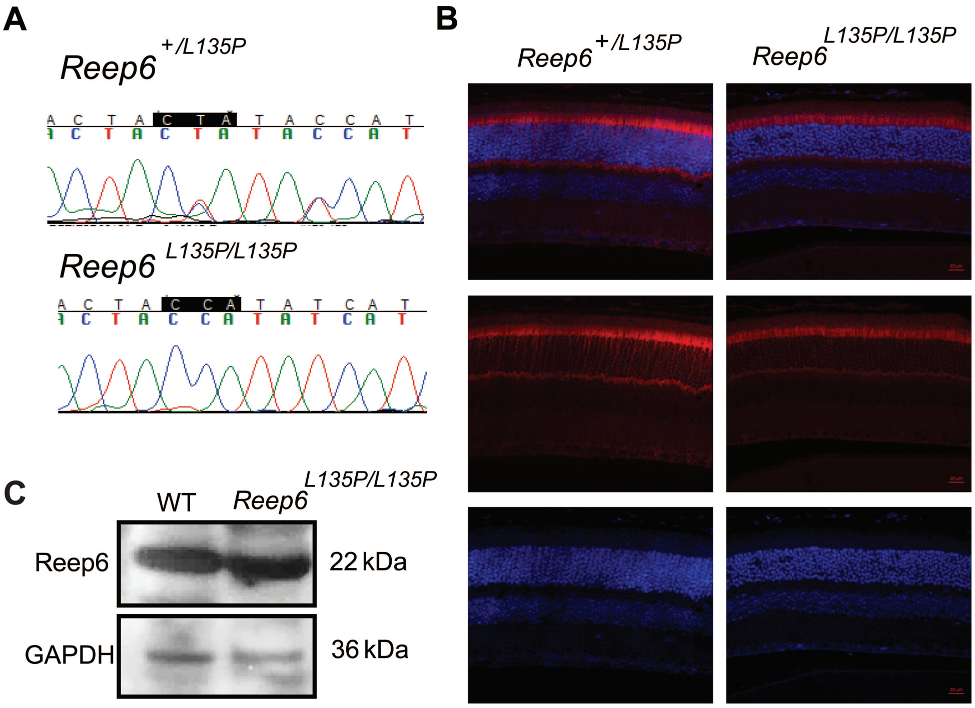

Figure S2. Genotyping of *Reep6* CRISPR/Cas9 Knock-in Mice and Reep6 Protein Expression in the Retina. (A) Chromatograms of CRISPR/Cas9 generated Reep6 heterozygous mice (*Reep6*$^{+/L135P}$) and knock-in (*Reep6*$^{L135P/L135P}$) mice. (**B**) Localization of Reep6 in *Reep6* knock-in mutant retina is unchanged compared to its localization in the *Reep6* heterozygous control retina. Scale bar: 20 µm (**C**) Western blot of retinal lysates from *Reep6* WT and *Reep6* knock-in shows that Reep6 protein levels are similar. GAPDH was used as loading control.

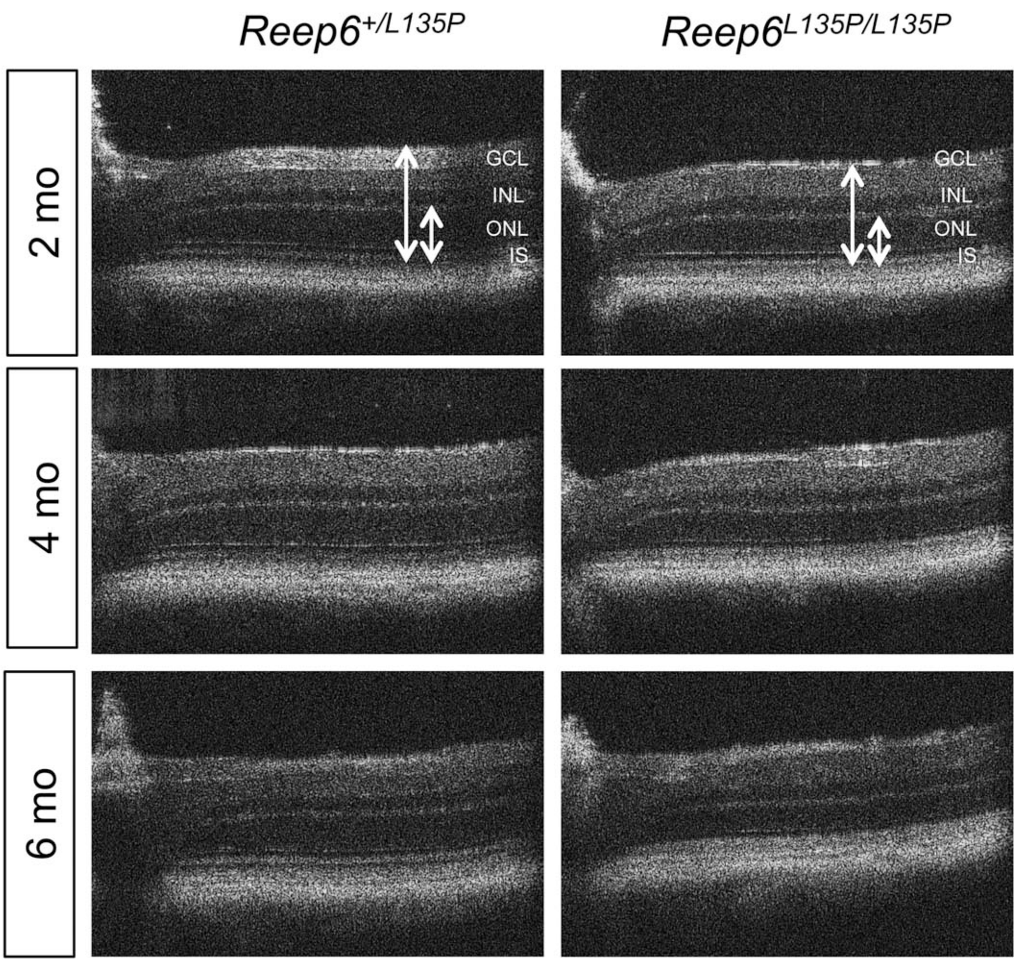

Figure S3. **SD-OCT analysis of *Reep6*$^{L135P/L135P}$ mice**. SD-OCT imaging showed thinning of the overall retina progressively (2 months to 6 months) and thinning of the ONL (arrow) in *Reep6*$^{L135P/L135P}$ mice retinas compared to littermate controls.

Chr	Coordinate	Ref	Obs	Function	Gene	Reason for exclusion	Consequence
13	32907359	A	C	nonsynonymous	BRCA2	Disease association	ENST00000544455.5: exon10:c.1744A>C, p.Thr582Pro
13	32912577	A	C	nonsynonymous	BRCA2		ENST00000544455.5: exon11:c.4085A>C, p.His1362Pro
11	67190894	C	T	stopgain	CARNS1		ENST00000445895.2: exon10:c.1675C>T, p.Gln559*
11	67191881	C	T	nonsynonymous	CARNS1	Benign prediction	ENST00000445895.2: exon10:c.2662C>T, p.Arg888Cys
20	62195499	C	T	nonsynonymous	HELZ2	LoF frequency in ExAC	ENST00000467148.1: exon9:c.4676G>A, p.Arg1559His
20	62198985	G	A	nonsynonymous	HELZ2		ENST00000427522.6 exon1:c.19C>T, p.Arg7Cys
3	195508451	G	T	nonsynonymous	MUC4	variants in cis	ENST00000463781.7: exon2:c.10000C>A, p.Pro3334Thr
3	195508453	C	T	nonsynonymous	MUC4		ENST00000463781.7: exon2:c.9998G>A, p.Ser3333Asn
3	195508454	T	C	nonsynonymous	MUC4		ENST00000463781.7: exon2:c.9997A>G, p.Ser3333Gly
19	1496339	T	C	nonsynonymous	REEP6	NA	ENST00000395484: exon4:c.404T>C, p.Leu135Pro
19	1496379	CG	C	frameshift	REEP6		ENST00000395484: exon4:c.448del, p.Ala150Profs*2
12	123957164	T	G	nonsynonymous	RILPL1	variants in cis	ENST00000376874.8: exon7:c.1133A>C, p.Glu378Ala
12	123957165	C	A	stopgain	RILPL1		ENST00000376874.8: exon7:c.1132G>T, p.Glu378*
10	99145008	A	T	nonsynonymous	RRP12		ENST00000370992.8: exon10:c.1123T>A, p.Tyr375Asn
10	99160207	G	C	nonsynonymous	RRP12	benign prediction	ENST00000370992.8: exon2:c.224C>G, p.Pro75Arg
11	19955532	C	T	nonsynonymous	NAV2	benign prediction	ENST00000528204.1: exon8:c.1811C>T, p.Ala604Val
11	19901474	Aagc	A	nonframeshift	NAV2		ENST00000528204.1: exon5:c.572_574del, p.191_192del

Table S1. Double heterozygous variants identified in individual A-II:1

Chr	Coordinate	Ref	Obs	Function	Gene	Consequence
1	27177711	A	G	nonsynonymous	ZDHHC18	ENST00000374142: exon6:c.925A>G, p.Thr309Ala
1	27238472	C	T	nonsynonymous	NR0B2	ENST00000254227: exon2:c.638G>A, p.Arg213His
1	152327526	T	A	nonsynonymous	FLG2	ENST00000388718: exon3:c.2736A>T, p.Gln912His
1	237872230	A	G	nonsynonymous	RYR2	ENST00000366574: exon69:c.9974A>G, p.Lys3325Arg
2	238736077	C	A	nonsynonymous	RBM44	ENST00000316997: exon12:c.2534C>A, p.Ala845Asp
2	241569895	C	G	nonsynonymous	GPR35	ENST00000438013: exon6:c.619C>G, p.Pro207Ala
2	242181952	C	T	nonsynonymous	HDLBP	ENST00000391975: exon17:c.2092G>A, p.Asp698Asn
2	242430588	C	T	nonsynonymous	FARP2	ENST00000264042: exon23:c.2617C>T, p.Pro873Ser
2	242814588	G	T	nonsynonymous	CXXC11	ENST00000343216: exon2:c.881G>T, p.Gly294Val
5	171765866	T	A	nonsynonymous	SH3PXD2B	ENST00000311601: exon13:c.2243A>T, p.Gln748Leu
6	18258544	T	C	nonsynonymous	DEK	ENST00000397239: exon3:c.238A>G, p.Ile80Val
10	71851666	G	C	nonsynonymous	H2AFY2	ENST00000373255: exon4:c.433G>C, p.Gly145Arg
10	96106258	G	A	nonsynonymous	NOC3L	ENST00000371361: exon11:c.1313C>T, p.Thr438Ile
15	33842470	G	A	nonsynonymous	RYR3	ENST00000389232: exon10:c.925G>A, p.Ala309Thr
15	48056050	G	A	nonsynonymous	SEMA6D	ENST00000316364: exon10:c.751G>A, p.Val251Met
15	50489851	G	T	nonsynonymous	SLC27A2	ENST00000267842: exon2:c.633G>T, p.Arg211Ser
16	30518143	C	T	nonsynonymous	ITGAL	ENST00000356798: exon21:c.2474C>T, p.Pro825Leu
16	31146747	G	A	nonsynonymous	PRSS8	ENST00000317508: exon1:c.73C>T, p.Arg25Trp
16	31193983	A	G	nonsynonymous	FUS	ENST00000254108: exon3:c.188A>G, p.Asn63Ser
16	31226444	C	T	nonsynonymous	TRIM72	ENST00000322122: exon2:c.385C>T, p.Leu129Phe
16	31373496	A	C	nonsynonymous	ITGAX	ENST00000268296: exon11:c.1187A>C, p.Glu396Ala
16	46993334	ATA A	-	Splice region	DNAJA2	ENST00000317089: c.775-6_775-3del
16	72821890	G	A	nonsynonymous	ZFHX3	ENST00000268489: exon10:c.10285C>T, p.Arg3429Cys
16	81219214	G	A	nonsynonymous	PKD1L2	ENST00000337114: exon11:c.1880C>T, p.Pro627Leu
16	82033626	T	C	nonsynonymous	SDR42E1	ENST00000328945: exon3:c.272A>G, p.Asn91Ser
16	87902985	G	A	nonsynonymous	SLC7A5	ENST00000261622: exon1:c.44C>T, p.Ala15Val
16	89925741	A	T	nonsynonymous	SPIRE2	ENST00000378247: exon9:c.1441A>T, p.Arg481Trp
17	48559747	A	C	nonsynonymous	RSAD1	ENST00000258955: exon4:c.770A>C, p.Tyr257Ser
17	48560058	G	A	nonsynonymous	RSAD1	ENST00000258955: exon5:c.895G>A, p.Val299Ile
17	48614425	C	T	nonsynonymous	EPN3	ENST00000268933: exon2:c.508C>T, p.Arg170Cys
17	48618328	C	T	nonsynonymous	EPN3	ENST00000268933: exon7:c.1154C>T, p.Thr385Ile
17	48618933	A	T	nonsynonymous	EPN3	ENST00000268933:

						exon9:c.1463A>T, p.Lys488Ile
17	48619437	-	CCGCCCCCAG CTGGCCTG	nonframeshift insertion	EPN3	ENST00000268933: exon10:c.1818_1819insCCGCCCCCAGCTGGCCTG, p.Ala606delinsAlaProProProAlaGlyLeu
17	48632895	C	T	nonsynonymous	SPATA20	ENST00000006658: exon17:c.2281C>T, p.Arg761Cys
17	74155492	C	T	nonsynonymous	RNF157	ENST00000269391: exon12:c.1228G>A, p.Val410Ile
19	1496625	-	C	frameshift insertion	REEP6	Genbank_EAW69491.1: Exon5:c.557dup, p.Val187Glyfs*13

Table S2. Homozygous rare variants identified in individual B-II:8

Family; Patient ID	Age/ gender/ country of origin	Age of onset/ symptoms	BCVA, logMAR (Snellen)	Refraction	Fundus	Age and summary of ERG	Other findings
EG76; A-II:1	35/M/Asia	5 nyctalopia,<br>18 field constriction	NA	NA	Peripheral retinal atrophy vessel attenuation bony spicules	20, only photopic responses tested, severely reduced	Goldmann visual fields reduced to 30 degrees with preserved temporal islands, early posterior subcapsular cataract
GC18419; B-II:8	54/M/Sudan	20 nyctalopia, 30 reduced fields, 40 reduced central vision	R 0.3 (20/40)<br>L 0.18 (20/30)	r +2.25/-1.25 x 75 L +2.5/-2 x 20	Attenuated vessels, mid-peripheral RPE atrophy, bone spicules and atrophic patches, CME	NA	Fields to confrontation 10 degrees, Ishihara 17/17 each eye, bilateral early cataract, anosmia age 48 years
GC20453; C-II:1	44/M/Turkey	10 nyctalopia, 37 reduced central vision	R 0.3 (20/40)<br>L 0.3 (20/40)	R pseudophakic, L -1.25/-0.75 x180	Optic disc pallor, attenuated vessels, mid-peripheral RPE atrophy, bone spicules and atrophic patches	NA	Fields to confrontation less than 10 degrees, Ishihara 17/17 each eye
GC20277; D-II:5	32/M/Iran	Early childhood nyctalopia, gradual field loss since	R 1.3 (20/400)<br>L 0.9 (20/160)	BE pseudophakic when assessed	Attenuated vessels, mid-peripheral RPE atrophy, minimal pigment change	Mid 20s, undetectable ERGs (scotopic and photopic)	Fields to confrontation 10-15 degrees, Ishihara 0/17 each eye
GC15672; E-II:1	29/M/India	14 nyctalopia, late teens fields loss	R 0.3 (20/40)<br>L 0.18 (20/30)	Myope	Attenuated vessels, mid-peripheral RPE atrophy and bone spicule hyperpigmentation, CME	15, PERG normal; rod ERG undetectable; delayed and profoundly reduced cone specific responses. Severe rod-cone dystrophy.	Fields to confrontation reduced to 30 degrees, early posterior subcapsular cataract

Table S3. Clinical Findings for *REEP6* affected individuals. Ages are indicated in years, M: male, F: female. NA: not available.
